# Preliminary treatment planning strategy for blood dose reduction in head and neck radiotherapy

**DOI:** 10.1002/acm2.70579

**Published:** 2026-04-13

**Authors:** Seohan Kim, Jieun Han, Wonmo Sung, Sebastian Tattenberg

**Affiliations:** ^1^ Department of Biomedical Engineering College of Medicine The Catholic University of Korea Seoul Republic of Korea; ^2^ Department of Medical Sciences Graduate School of The Catholic University of Korea Seoul Republic of Korea; ^3^ CMC Institute for Basic Medical Science The Catholic Medical Center of The Catholic University of Korea Seoul Republic of Korea; ^4^ School of Natural Sciences Laurentian University Sudbury Ontario Canada; ^5^ Life Sciences Division TRIUMF Vancouver British Columbia Canada

**Keywords:** blood dose, blood vessel‐sparing RT, head and neck cancer, planning study, treatment plan optimization

## Abstract

**Background:**

Radiation dose to circulating blood, a surrogate for circulating lymphocyte damage by radiotherapy (RT), has emerged as a factor predicting treatment outcomes, including overall survival, tumor recurrence, and lymphopenia occurrence. This study aimed to investigate strategies for reducing circulating blood dose in head and neck cancer (HNC) patients by comparing treatment plans, the implementation of which varied by modality and blood vessel‐sparing (BVS) approach.

**Materials and Methods:**

Using a publicly available dataset, 20 HNC patients were randomly selected and a total of 120 treatment plans were generated using RayStation. For each of the two modalities, including intensity‐modulated radiation therapy (IMRT) and intensity‐modulated proton therapy (IMPT), three planning strategies were evaluated: a conventional plan (Conv) and two blood vessel‐sparing plans with mean dose constraints targeting 90% and 80% of the conventional vessel dose (BVS‐90% and BVS‐80%, respectively). Blood dose was estimated using the HEDOS simulation framework.

**Results:**

IMPT reduced circulating blood dose compared to IMRT, lowering the mean blood dose by 21.5% and D_90%_ by 31.8%. The BVS‐80% constraint further decreased the mean blood dose by 12.5% for IMPT and 12.6% for IMRT, while the intermediate BVS‐90% constraint yielded reductions of 8%. These reductions were achieved without clinically meaningful compromise in target coverage or increase in dose to other organs‐at‐risk (OARs). Monitor unit analysis revealed no significant increase in delivery complexity with BVS planning.

**Conclusions:**

In this preliminary planning study, incorporating blood‐rich structures as OARs and adopting IMPT effectively reduced blood dose. Integrating blood dose considerations into RT planning is readily implementable within existing clinical workflows to minimize lymphocyte damage.

## 1 INTRODUCTION

Radiation‐induced lymphopenia (RIL) is a common adverse side effect occurring in patients receiving radiotherapy (RT) regardless of tumor site.^1^, ^2^, ^3^ This is attributed to the high radiosensitivity of lymphocytes, with studies indicating that even low radiation doses (0.125 Gy) can trigger apoptosis.^4^, ^5^ Managing lymphocyte depletion during RT is crucial, given that lymphocytes play a central role in immune responses and are closely associated with clinical outcomes in cancer treatment.^6^ Specifically, previous studies have demonstrated correlations between severe RIL and reduced overall survival as well as increased tumor recurrence.^1^, ^7^, ^8^ Moreover, severe RIL has been associated with poor treatment outcomes in patients receiving immunotherapy, as the depletion of lymphocyte populations may compromise immune‐mediated tumor surveillance and the synergistic effects of immunotherapy combined with RT.^2^, ^6^, ^7^, ^8^, ^9^

To predict blood cell depletion during RT, several surrogate methodologies have been developed: integrated body dose, effective dose to immune cells (EDIC), dose to recirculating lymphocytes (LymphoDose), flow and irradiation personalized approach (FLIP), and hematological dose (HEDOS).^10^, ^11^, ^12^, ^13^, ^14^, ^15^ Among these, the HEDOS framework provides realistic spatiotemporal blood dynamics across the whole body, allowing for the calculation of the circulating blood dose regardless of the treatment site.^13^ This, in turn, enables quantification of circulating blood dose based on the dose delivered to blood‐related organs across different treatment settings. Furthermore, clinical applicability of blood dose estimates from HEDOS has been demonstrated through its association with clinical data regarding lymphocyte depletion.^16^, ^17^ However, since HEDOS hasn't been used in plan optimization yet, its integration into treatment planning studies could offer valuable insights into strategies for reducing circulating blood dose.

Blood dose reduction in RT can be achieved through the appropriate selection of the treatment modality and relevant dose constraints. In terms of treatment modality, proton therapy effectively reduces the low‐dose bath, which is strongly associated with circulating lymphocyte damage.^7^, ^18^ Furthermore, high‐dose‐rate RT such as hypofractionation has been investigated for its superiority to minimize the volume of irradiated circulating blood by significantly reducing beam delivery time.^19^ With respect to plan optimization, proper dose constraints for blood‐rich organs may help spare circulating blood. Given that each organ has a distinct blood flow and volume, their contributions to the overall blood dose vary accordingly. Therefore, careful consideration of these factors in RT planning is essential to enhance immune preservation.

However, despite the growing recognition of its importance, blood vessel‐sparing RT has yet to be incorporated into standard RT planning protocols. This gap may stem from the limited quantitative investigations assessing the impact of blood vessel‐sparing strategies on circulating blood dose in the context of clinical treatment planning. To address this, the present study aims to conduct a preliminary treatment planning analysis evaluating the feasibility and dosimetric impact of blood vessel‐sparing strategies on circulating blood dose in head and neck cancer (HNC) patients. Specifically, using data from HNC patients, we investigate optimization strategies through additional blood vessel‐sparing dose constraints in photon and proton treatment planning.

## 2 MATERIALS AND METHODS

### 2.1 Patient dataset

The “Head‐Neck‐PET‐CT” dataset from the HECKTOR 2025 challenge (https://hecktor25.grand‐challenge.org/dataset/), a publicly available resource, contains computed tomography (CT) images and RT contour data for 298 HNC patients.^20^ In this study, patient selection required that all target volumes and all organ‐at‐risks (OARs) not reliably delineable using the selected auto‐segmentation tool had been manually contoured in the treatment planning CT. From the dataset of patients that fulfilled the above criteria, 20 patients were selected via random number generation. To ensure non‐preferential selection with respect to criteria, target volume as well as blood vessel position relative to the location of the irradiated targets were not considered. The selected cohort predominantly comprised oropharyngeal primary sites cases (*n* = 18), with hypopharyngeal and unknown cases (Table 1). Using this dataset, automatic contouring was performed with TotalSegmentator, following the same approach as in the previous study by Tattenberg et al.^21^, ^22^ In line with their framework for blood dose calculation, which included structures such as fat, skeletal muscle, skeleton, thyroid, and major blood vessels (brachiocephalic veins, brachiocephalic trunk, common carotid arteries, internal carotid arteries, internal jugular veins, pulmonary artery, subclavian arteries, and superior vena cava), this study extended the segmentation scope to include lymph nodes. Additionally, the brainstem was contoured as part of the conventional OAR set. These additional structures were generated using the deep learning segmentation feature in RayStation version 2024b (RaySearch Laboratories, Stockholm, Sweden).

**TABLE 1:** Cohort characteristics.

Characteristic	Number
Sex	
Male	15
Female	5
Age	
40–49	1
50–59	9
60–69	6
70–79	3
80–89	1
TNM stage	
II	1
III	1
IV	17
N/A	1
Primary tumor site	
Oropharynx	18
Hypopharynx	1
Unknown	1
PTV 1 volumes, cm^3^	
100–199	2
200–299	5
300–399	7
400–499	4
500–599	2
PTV 2 volumes, cm^3^	
0–99	1
100–199	8
200–299	8
300–399	1
400–499	1
500–599	1

Abbreviations: D_max_, maximum dose; D_mean_, mean dose; Dn%, the minimum dose received by at least n% of the volume; DVH, dose‐volume histogram; PTV, planning target volume.

### 2.2 Treatment planning

Comparative RT treatment plans were generated using RayStation version 2024b for two modalities: intensity‐modulated radiation therapy (IMRT) and intensity‐modulated proton therapy (IMPT). IMRT plans were generated using a 6 MV photon beam with nine field arrangements at 0°, 40°, 80°, 120°, 160°, 200°, 240°, 280°, and 320°, utilizing dynamic multi‐leaf collimation on a TrueBeam STX system (Varian Medical Systems, Palo Alto, CA, USA). For IMPT, pencil‐beam scanning was employed with four beam angles (50°, 140°, 188°, and 325°) delivered via the ProteusONE system (Ion Beam Applications, Louvain‐La‐Neuve, Belgium). Each case included two PTVs, PTV1 for the primary tumor and PTV2 for elective nodal irradiation. Prescription doses were set at 60 Gy for PTV1 and 52 Gy for PTV2, delivered in 33 fractions. PTV volumes are reported in Table 1 and a visual explanation is represented in Figure SA1.

For each modality, three treatment plans were generated: a conventional plan using established OAR dose constraints, and two blood vessel‐sparing (BVS) plans incorporating additional dose constraints applied to major vascular structures, specifically large arteries and large veins.^23^, ^24^ Given that a substantial fraction of circulating blood passes through large vessels within typical HNC dose distributions, the BVS plans imposed mean dose constraints to these vessels. Due to inter‐patient variability in baseline dose distribution, applying a uniform absolute dose constraint across all cases was not appropriate for fair comparison. Instead, patient‐specific constraints were derived, targeting a mean dose to large vessels at 80 or 90% of the corresponding value in the conventional plan. The target and OAR dose‐volume constraints for all treatment plans are summarized in Table 2.

**TABLE 2:** The dose‐volume objectives utilized.

Structure	Applied to plan	Dose‐volume objectives used	Relative priority
PTV 1	All plans	D_0.1%_ < 60.6 Gy	1000
		D_98%_ > 59.4 Gy	
PTV 2	All plans	D_0.1%_ < 52.52 Gy	1000
		D_98%_ > 51.48 Gy	
Brain	All plans	D_max_ ≤ 60 Gy	100
Brain stem	All plans	D_max_ < 54 Gy	100
Esophagus	All plans	D_mean_ < 34 Gy	100
Larynx	All plans	D_mean_ < 44 Gy	100
Mandible	All plans	D_mean_ < 60 Gy	100
Oral cavity	All plans	D_mean_ < 45 Gy	100
Optic chiasm	All plans	D_max_ < 55 Gy	100
Optic nerve	All plans	D_max_ < 55 Gy	100
Parotid gland	All plans	D_mean_ ≤ 20 Gy	100
Spinal cord	All plans	D_max_ < 50 Gy	100
Large arteries	Blood vessel‐sparing plans	90% or 80% of $D_{\mathrm{mean}}^{\mathrm{Large}\;\mathrm{arteries}}$ in plan without blood vessel‐sparing^a^	200
Large veins	Blood vessel‐sparing plans	90% or 80% of $D_{\mathrm{mean}}^{\mathrm{Large}\;\mathrm{veins}}$ in plan without blood vessel‐sparing^a^	200

Abbreviations: D_max_, maximum dose; D_mean_, mean dose; Dn%, the minimum dose received by at least n% of the volume; DVH, dose‐volume histogram; PTV, planning target volume.

^a^ A mean dose to large vessels at 90% or 80% of the corresponding non‐blood vessel‐sparing plan.

**FIGURE 1:**
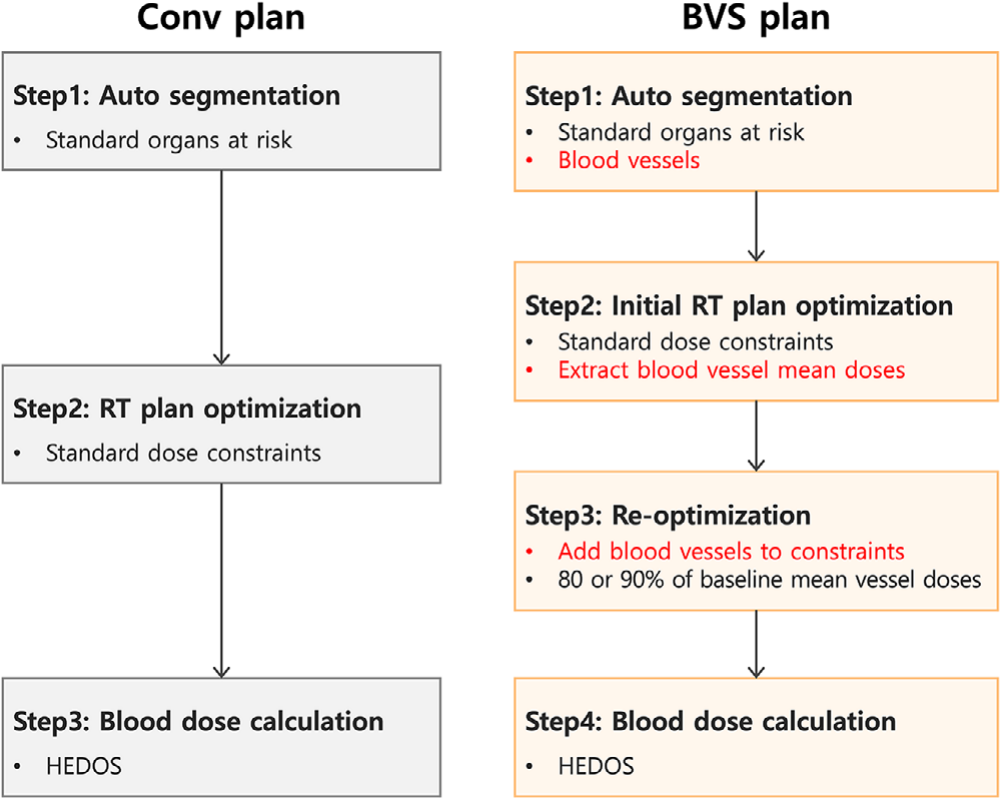
Comparison of radiotherapy (RT) treatment planning workflows between conventional (Conv) and blood vessel‐sparing (BVS) approaches. The Conv (left) and BVS plan (right) follow a two‐step and four‐step process, respectively.

To summarize, six treatment plans per patient were generated for IMRT with blood vessel‐sparing (IMRT‐BVS‐80% and IMRT‐BVS‐90%), IMRT without blood vessel‐sparing (IMRT‐Conv), IMPT with blood vessel‐sparing (IMPT‐BVS‐80% and IMPT‐BVS‐90%), and IMPT without blood vessel‐sparing (IMPT‐Conv), yielding a total of 120 treatment plans across 20 patients. Following dose optimization, dose‐volume histograms (DVHs) were calculated for both target volumes and OARs. The workflow to generate each plan is illustrated in Figure 1, which compares the conventional planning approach with the blood vessel‐sparing plan.

### 2.3 Evaluation metrics for treatment plan comparability

To ensure a fair comparison between treatment plans, each optimized plan was designed to achieve comparable tumor coverage, irrespective of the treatment modality or blood vessel‐sparing strategy. Plan quality was assessed using the homogeneity index (HI) and conformity index (CI) to evaluate the dose distribution within the PTV.^25^ The uniformity of the dose distribution within the target volume was assessed using the HI, defined as:

$$HI=\frac{D_{5\%}}{D_{95\%}}$$

where $D_{n\%}$ corresponds to the dose in the DVH at n% of the target volume. HI values closer to unity indicate more homogeneous dose distributions within the target. The spatial conformity of the high‐dose region relative to the target was evaluated using the CI, calculated as:

$$CI=\frac{\mathrm{Volume}\;\mathrm{within}\;\mathrm{the}\;95\%\;\mathrm{isodose}}{\mathrm{Volume}\;\mathrm{of}\;\mathrm{PTV}}$$

This metric represents the ratio of the total tissue volume receiving at least 95% of the prescribed dose to the PTV volume, with an ideal CI value approaching unity. For each plan, HI and CI values were derived from the PTV DVHs, ensuring consistency in dose coverage across different planning approaches.

### 2.4 Calculation of dose‐volume histograms for circulating blood

HEDOS was utilized to estimate the radiation dose absorbed by circulating blood.^13^, ^15^ HEDOS simulates the spatiotemporal trajectory of blood particles throughout the body based on a whole‐body blood flow model from the International Commission on Radiological Protection (ICRP) report 89.^26^ In brief, the framework discretizes the total blood volume into a large number of computational particles, each representing a small volume of blood. At every time step, each particle is probabilistically assigned to an organ according to the organ‐specific fractional blood flow and transit time distributions derived from the ICRP model. During the simulated beam delivery time, particles residing in organs within the radiation field accumulate dose sampled from the organ‐level DVH. By tracking the cumulative dose to all particles across the full treatment phase, HEDOS constructs a circulating blood DVH that accounts for both the spatial dose distribution and the temporal dynamics of blood circulation.

To accurately simulate blood flow dynamics, 10^6^ blood particles were modeled with a time step of 0.05 s. Total blood volume and cardiac output were determined based on sex‐specific reference values from ICRP report 89, where male patients were assigned 5.3 L of total blood volume and 6.5 L/min of cardiac output, while female patients were assigned a total blood volume of 3.9 L and a cardiac output of 5.9 L/min.

For each treatment plan, circulating blood DVHs were simulated using organ‐specific DVHs and treatment parameters as inputs. Beam delivery time for an RT field was defined as 40 and 30 s for IMRT and IMPT, respectively. Although HEDOS is capable of incorporating dose distributions from all organs included in the blood flow model, this study focused on organs within the HNC RT field. The following anatomical structures were considered in blood dose estimation: aorta, bone, brain, bronchi, fat, large arteries, large veins, lungs, lymph nodes, skeletal muscles, skin, stomach, esophagus, superior vena cava, thyroid, and the residual body volume excluding other delineated organs.

### 2.5 Statistical analysis

To assess the differences in parameters of interest between different planning approaches, appropriate statistical tests were employed based on the comparison structure. For the evaluation of three constraint levels (Conv, BVS‐90%, BVS‐80%) within each modality, the Friedman test was conducted, followed by post‐hoc Wilcoxon signed‐rank tests with Bonferroni correction when significant differences were detected. A significance threshold of *p* < 0.05 was applied for all statistical analyses.

## 3 RESULTS

### 3.1 Treatment plan comparability

**FIGURE 2:**
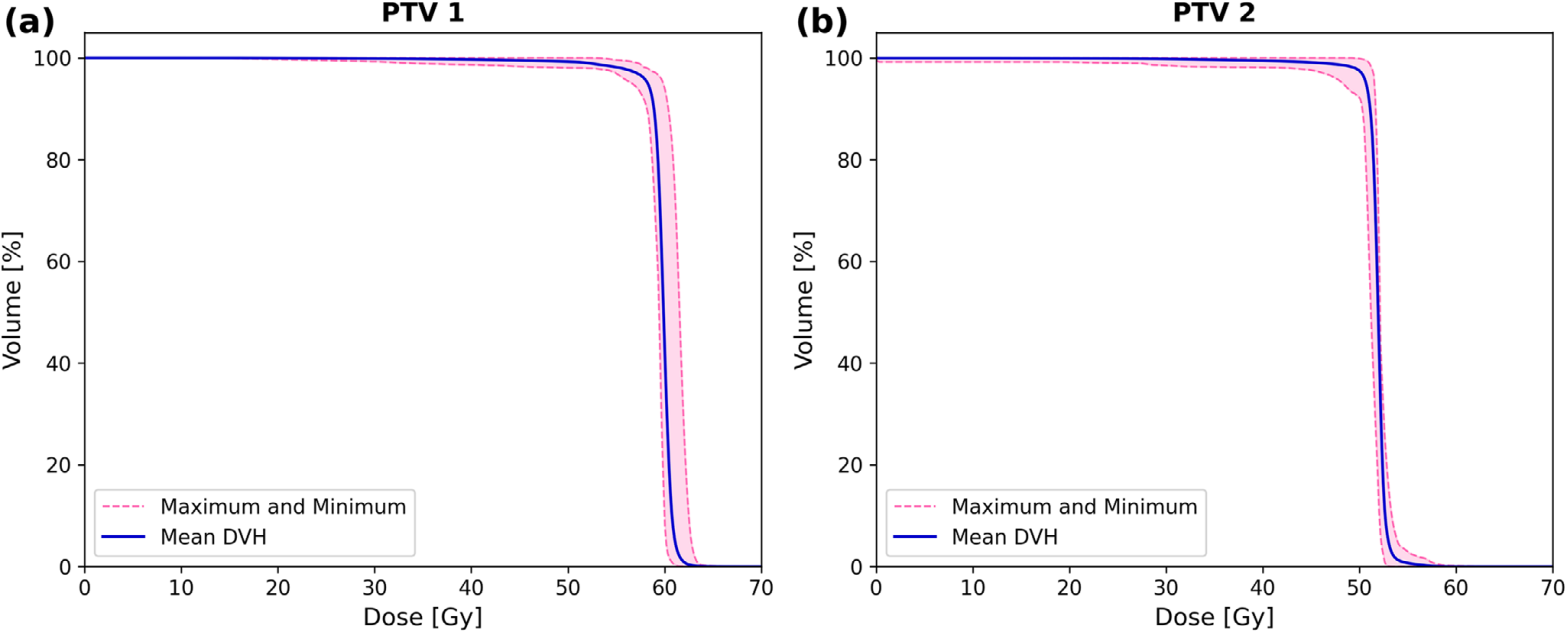
Dose‐volume histograms (DVHs) for the planning target volumes (PTVs). (a) DVH for PTV1 and (b) DVH for PTV2, illustrating the maximum and minimum DVHs (dashed magenta lines), and the mean DVH of all plans (solid blue line). The consistency of the dose distributions across different RT plans confirms adequate target coverage with minimal deviations.

All PTV DVHs across RT treatment plans demonstrated overall consistency in dose distributions within PTVs (Figure 2). As summarized in Table 3, statistically significant differences in HI and CI were detected across several comparisons (*p* < 0.05). Post‐hoc Wilcoxon signed‐rank test revealed that these differences were primarily observed between Conv and BVS‐80% plans, whereas Conv versus BVS‐90% comparisons were largely not significant in IMRT‐PTV1 and IMPT‐PTV1. Despite statistical significance in several comparisons, the absolute variations in HI and CI were minimal. Across all conditions, HI values remained within 1.02–1.10, and CI consistently exceeded 96%, indicating clinically acceptable dose homogeneity and spatial conformity. These findings suggest that while minor statistical differences were detected with more rigorous constraints to vessels, overall target coverage and plan quality were maintained.

**TABLE 3:** Target coverage evaluation.

Index	Conv	BVS‐90%	BVS‐80%	*p* value	Conv vs. BVS‐90%^a^	Conv vs. BVS‐80%^a^	BVS‐90% vs. 80%^a^
IMRT‐PTV 1							
HI (range)	1.04 (1.03–1.07)	1.03 (1.02–1.07)	1.05 (1.02–1.08)	**<0.001**	0.228	0.072	**0.0002**
CI (range)	97.74 (94.89–98.47)	97.62 (95.43–98.16)	96.99 (94.40–98.04)	**0.024**	1.000	**0.013**	**0.007**
IMRT‐PTV2							
HI (range)	1.02 (1.02–1.04)	1.03 (1.02–1.06)	1.04 (1.02–1.10)	**<0.001**	**0.0001**	**<0.0001**	**<0.0001**
CI (range)	98.77 (97.40–99.96)	98.43 (96.16–99.43)	97.56 (93.08–98.57)	**<0.001**	**<0.0001**	**0.0003**	**<0.0001**
IMPT‐PTV 1							
HI (range)	1.05 (1.03–1.07)	1.05 (1.04–1.07)	1.05 (1.04–1.08)	**0.043**	0.995	0.100	0.110
CI (range)	97.15 (95.50–98.34)	97.56 (95.72–99.09)	96.81 (95.34–98.36)	**0.011**	0.079	0.089	**0.001**
IMPT‐PTV2							
HI (range)	1.05 (1.03–1.06)	1.05 (1.03–1.07)	1.06 (1.03–1.08)	**0.001**	0.186	**0.002**	0.607
CI (range)	98.66 (97.82–99.38)	98.28 (97.00–98.66)	97.42 (95.91–98.58)	**<0.001**	**0.002**	**<0.0001**	**<0.0001**

*Note*: *p* values were derived using Friedman test, followed by ^a^post‐hoc Wilcoxon signed‐rank test.

Abbreviations: BVS, blood vessel‐sparing plan; CI, conformity index; Conv, non‐blood vessel‐sparing (conventional) plan; HI, homogeneity index; IMPT, intensity‐modulated proton therapy; IMRT, intensity‐modulated radiation therapy; PTV, planning target volume.

**FIGURE 3:**
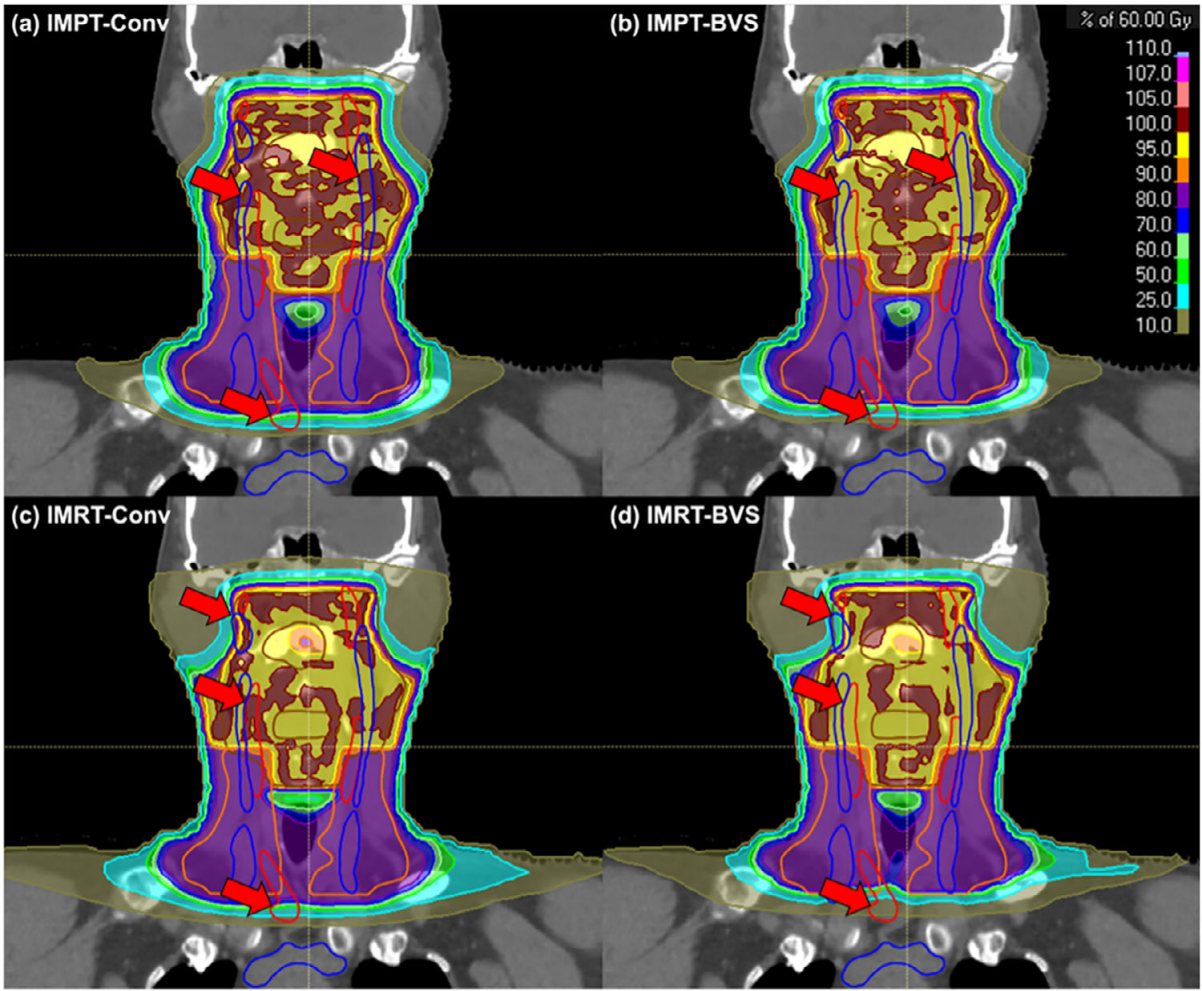
The sample coronal dose distributions for each treatment plan. Large arteries and veins are overlaid with red and blue solid contours, respectively. Red arrows indicate regions where the dose is reduced by the blood vessel‐sparing (BVS) plan. (a) IMPT‐Conv: conventional intensity‐modulated proton therapy. (b) IMPT‐BVS: blood vessel–sparing intensity‐modulated proton therapy. (c) IMRT‐Conv: conventional intensity‐modulated photon radiotherapy. (d) IMRT‐BVS: blood vessel–sparing intensity‐modulated photon radiotherapy. Dose colorwash displays percent of prescribed 60 Gy (see legend).

Regarding OARs, statistically insignificant differences were observed in D_mean_ or D_max_ for the majority of OARs across Conv, BVS‐90%, and BVS‐80% plans within each modality, including the mandible, oral cavity, larynx, and bilateral parotid glands (Friedman test; all *p* > 0.05 in at least one modality). Brain and brainstem D_max_ showed statistically significant increases at the BVS‐80% level in IMRT (brain: *p* = 0.008; brainstem: *p* = 0.011). However, these increases were modest in absolute magnitude and all values remained within or near the respective objectives. The esophagus was the only conventional OAR to exhibit a significant and systematic change, with D_mean_ decreasing progressively from Conv to BVS‐80% in both modalities (both *p* < 0.001). This reduction represents a concurrent benefit arising from the anatomical adjacency of the esophagus to the targeted cervical vessels. Large arteries and large veins also showed the expected dose reductions from Conv to BVS‐80% (all *p* < 0.001). As illustrated in Figure 3, the application of blood vessel constraints reduces high‐dose exposure to major blood vessels in BVS plans compared to Conv plans. Detailed DVH metrics for OARs are reported in Table SA1.

### 3.2 Effects of treatment modality on blood dose reduction

**FIGURE 4:**
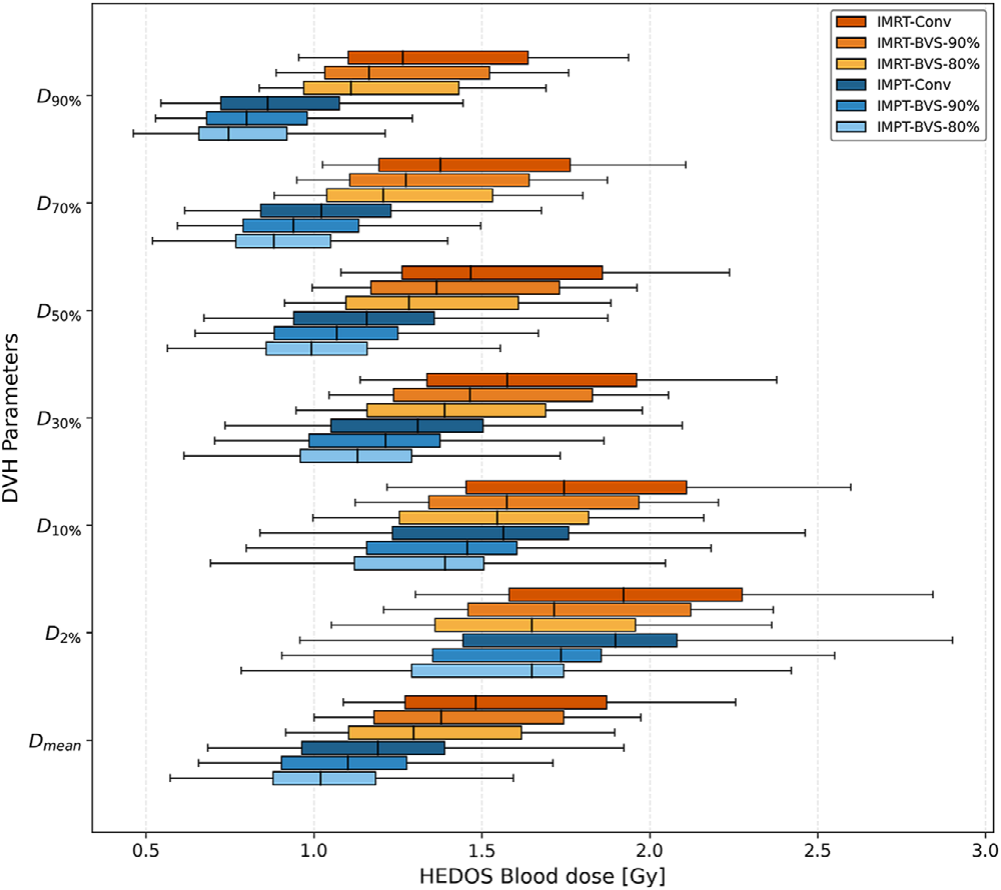
Comparison of HEDOS blood dose‐volume histogram (DVH) parameters for six radiotherapy (RT) treatment plans. DVH parameters of circulating blood, including mean dose ($D_{\mathrm{mean}}$) and minimum dose received by at least 90%, 70%, 50%, 30%, 10%, and 2% of the circulating blood volume ($D_{90\%}$, $D_{70\%}$, $D_{50\%}$, $D_{30\%}$, $D_{10\%}$, and $D_{2\%}$, respectively). Box plots represent the interquartile range with solid line indicating median values of each metric.

The dosimetric comparison between IMPT and IMRT revealed a consistent reduction in circulating blood dose with IMPT across all points on the DVH. Reported values represent the median with the range of each metric. On average, IMPT resulted in a 21.5% reduction in the D_mean_, decreasing from 1.482 (1.087–16.483) Gy for IMRT to 1.198 (0.684–16.235) Gy for IMPT. In the low‐dose range, IMPT demonstrated substantial reductions as well. The minimum dose received by at least 90% of the blood volume (D_90%_) decreased by 31.8%, from 1.284 (0.954–15.649) Gy for IMRT to 0.862 (0.544–14.774) Gy for IMPT. Likewise, the fraction of blood volume receiving at least 0.9 Gy (V_0.9 Gy_) was 24% lower in IMPT 85.7 (4.5–100)% compared to IMRT 100 (97.3–100)%. The dosimetric advantages of IMPT extended beyond the low‐dose spectrum into higher dose regions. The D_2%_ was reduced by 5.8% on average from 1.928 (1.302–17.847) Gy for IMRT to 1.909 (0.958–18.646) Gy for IMPT. Further detailed values for these parameters are provided in Figure 4 and Table SA2.

### 3.3 Effects of dose constraints for blood vessel sparing on blood dose reduction

Within the IMPT modality, the Friedman test revealed statistically significant differences among the three constraint levels across all blood DVH metrics (*p* < 0.001). The BVS‐80% reduced the median D_mean_ from 1.198 (0.684–16.235) Gy for IMPT‐Conv to 1.059 (0.572–16.218) Gy, corresponding to a mean per‐patient reduction of 12.5%. The intermediate BVS‐90% achieved a mean per‐patient reduction of 8.2%, lowering the median D_mean_ to 1.101 (0.657–16.204) Gy. The reduction extended consistently across the DVH spectrum. D_90%_ decreased from 0.862 (0.544–14.774) Gy for IMPT‐Conv to 0.766 (0.462–14.751) Gy for IMPT‐BVS‐80% (12.0% mean reduction) and 0.799 (0.529–14.743) Gy for IMPT‐BVS‐90% (7.6% mean reduction). The stepwise comparison between BVS‐90% and BVS‐80% also yielded statistically significant additional reductions across all DVH metrics (*p* < 0.02).

In IMRT, the BVS strategies demonstrated significant reduction for all DVH metrics (Friedman test, *p* < 0.001). The median D_mean_ decreased to 1.308 (0.915–16.446) Gy for IMRT‐BVS‐80% from 1.482 (1.087–16.483) Gy for IMRT‐Conv, representing a mean per‐patient reduction of 12.6%. The BVS‐90% yielded a 7.5% mean per‐patient reduction, with a median D_mean_ of 1.379 (1.001–16.457) Gy. A comparable trend was observed in D_90%_, which dropped from 1.284 (0.954–15.650) Gy to 1.118 (0.837–15.605) Gy (BVS‐80%, 12.2% mean reduction) and 1.190 (0.887–15.619) Gy (BVS‐90%, 7.2% mean reduction). The pairwise comparison between BVS‐90% and BVS‐80% was statistically significant for all DVH metrics (*p* < 0.003).

These results demonstrate that blood dose reduction was consistently observed at both constraint levels and across both modalities, with the magnitude of reduction proportional to the vessel‐sparing level (Figure 4). For BVS‐80%, per‐patient mean reductions ranged from 12.0% to 13.2% across DVH metrics, while BVS‐90% yielded reductions of 7.2% to 8.8% (Table SA2). The stepwise comparison between BVS‐90% and BVS‐80% showed statistical significance, confirming that tighter vessel‐sparing constraints provide additional measurable benefit. It should be noted that the hypopharyngeal case exhibited a substantially higher blood dose (D_mean_ > 16 Gy) compared to the oropharyngeal cases (–1.5 Gy) and showed negligible reduction from BVS constraints (Figure SA2). Overall, incorporating large vessels as OARs effectively reduced circulating blood dose regardless of modality, with the lowest blood dose consistently observed in IMPT‐BVS‐80%.

## 4 DISCUSSIONS

To investigate the effects of RT treatment plans on circulating blood dose, the present study conducted a preliminary treatment planning analysis in which blood dose optimization was conducted based on both treatment modality (IMRT and IMPT) and the inclusion of additional planning constraints for large blood vessels at two threshold levels (BVS‐90% and BVS‐80%). Our findings demonstrated that IMPT effectively suppressed the low‐dose bath and consistently reduced circulating blood dose compared to IMRT, with a 21.5% decrease in D_mean_ from 1.482 Gy to 1.198 Gy and a 31.8% reduction in D_90%_ from 1.284 Gy to 0.862 Gy. The introduction of blood vessel‐sparing constraints further enhanced blood dose reductions, achieving a 12.5% decrease in D_mean_ for IMPT and a 12.6% reduction for IMRT at the BVS‐80%. While IMPT proved to be a highly effective modality for mitigating blood dose, the majority of HNC patients receive IMRT. Importantly, the results suggest that even within IMRT, circulating blood dose can be minimized via the consideration of appropriate OARs in which blood‐rich structures are included. While these findings are derived from a limited cohort and a retrospective planning study without clinical outcome validation, they provide an initial framework for incorporating blood dose considerations into RT planning.

The selection of the vessel‐sparing constraint threshold is an important consideration for clinical implementation. Since tumor sites, PTV volumes, and target‐to‐vessel spatial relationships vary across patients, we employed patient‐specific relative constraints rather than fixed absolute dose limits to ensure consistent applicability. To investigate whether the reduction of blood dose depends on the specific threshold, we evaluated two constraint levels (BVS‐90% and BVS‐80%) as a sensitivity analysis. The BVS‐90% constraint achieved a meaningful 8%–10% reduction in mean blood dose, while the more aggressive BVS‐80% constraint yielded approximately 13% reduction for both modalities. These results demonstrate that blood dose decreases proportionally with the applied constraint level, indicating that the conclusions of this study are not contingent upon any single threshold value. However, there is a practical limit to this proportional reduction, as aggressive constraints compromise PTV coverage. At the BVS‐80%, modest but statistically significant changes in HI and CI were observed. During treatment planning, constraints beyond this level led to rapid deterioration of PTV coverage. The 80% threshold therefore represents a practical lower bound under the current planning framework, beyond which target coverage becomes clinically unacceptable. Taken together, these findings suggest that the best balance between blood dose reduction and target coverage depends on institutional preferences and patient‐specific anatomy.

**FIGURE 5:**
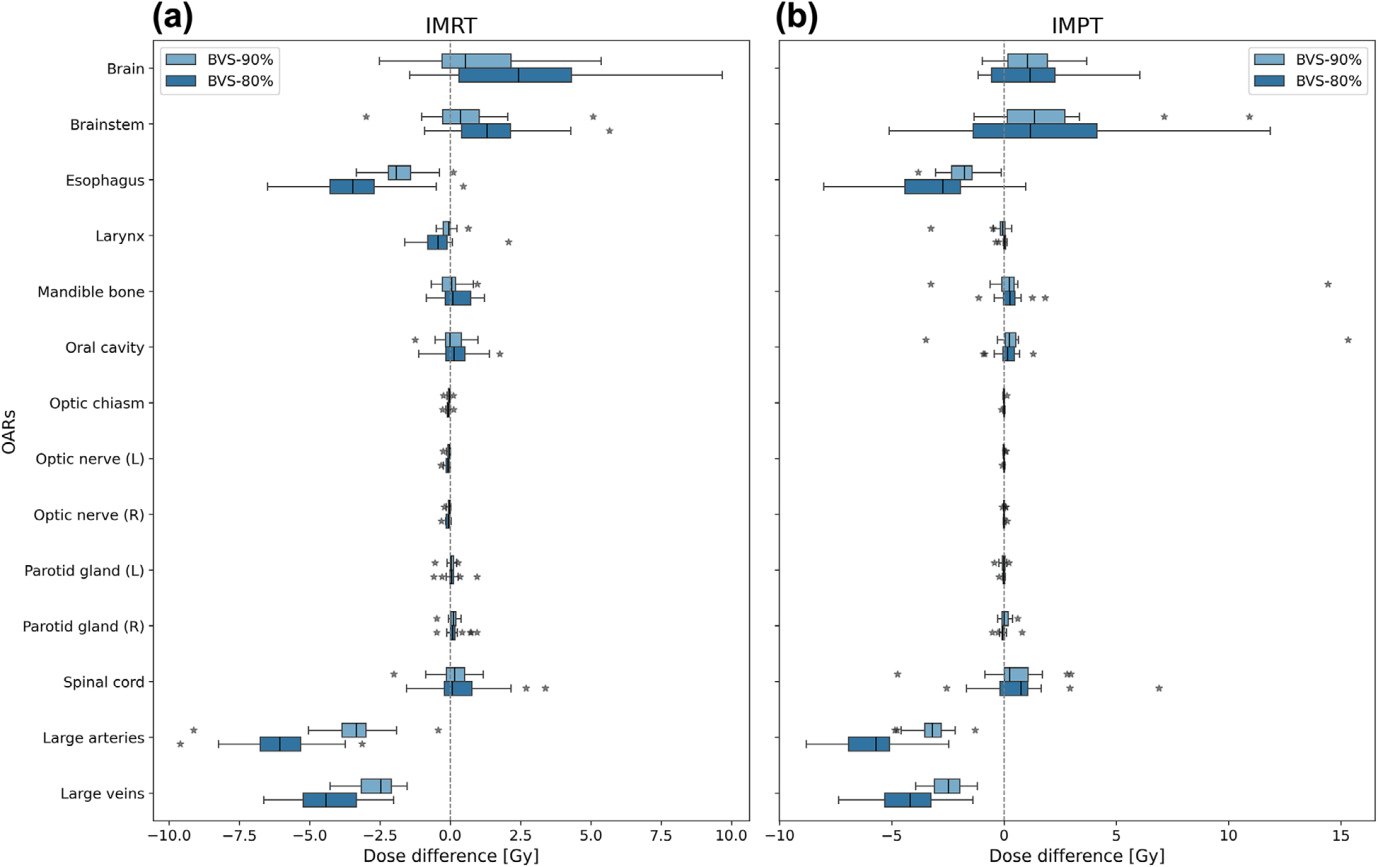
Dose differences between blood vessel‐sparing (BVS) and conventional (Conv) plans for organs‐at‐risk (OARs). The differences were calculated by subtracting Conv dose from BVS dose for each patient. D_max_ was used for brain, brainstem, optic chiasm, optic nerves, and spinal cord, while D_mean_ was employed for the remaining structures. Results are shown separately for (a) intensity‐modulated radiotherapy (IMRT) and (b) intensity‐modulated proton therapy (IMPT). Two constraint levels are compared: BVS‐90% (light blue) and BVS‐80% (dark blue). Box plots represent the interquartile range with whiskers indicating the full range of values, excluding outliers. Negative values indicate dose reduction in BVS plans relative to Conv plans.

The impact of blood vessel‐sparing constraints on OAR doses was further evaluated by comparing the dosimetric differences between BVS and Conv plans (Figure 5). Among all OARs, the brain and brainstem exhibited the most notable changes, with increased D_max_ in BVS plans relative to Conv plans. Since the D_mean_ of BVS plans did not differ significantly from that of Conv plans, the dose spared from large vessels appears to be redistributed toward adjacent structures that share the same high‐dose region. The brainstem and inferior brain border are anatomically situated between the PTVs and the major cervical vasculature, in which the redistributed dose is preferentially deposited. Furthermore, because D_max_ rather than D_mean_ serves as the planning objective for these structures, the apparent change is amplified relative to that of organs evaluated by D_mean_. Conversely, the esophagus demonstrated a consistent decrease in mean dose under BVS constraints, as expected given its proximity to the constrained vessels. For the remaining OARs, the absolute dosimetric differences between BVS and Conv plans were small, implying that the dose redistribution effect is spatially confined to structures near the constrained vessels. These patterns suggest that future studies should examine how OAR‐specific trade‐offs associated with the BVS strategy vary across individual cases.

Our strategy that imposes dose constraints on large vessels can achieve effective blood vessel‐sparing without significantly elevating the complexity of HNC RT treatment plans. More sophisticatedly modulated beams may inevitably lead to an increase in monitor units (MU) and therefore also both treatment time and peripheral (i.e., out‐of‐field) dose.^27^, ^28^, ^29^ While intensity modulation inherently affects MUs, appropriate blood vessel sparing did not impose an additional burden in this regard. As shown in Table 4, MU numbers remained statistically comparable between Conv and BVS plans (Wilcoxon signed rank test, *p* > 0.05), indicating that the applied constraints were uninfluential to excessive modulation complexity. This finding is of particular clinical relevance, as it implies that blood vessel‐sparing techniques can be integrated into RT planning without compromising delivery efficiency or increasing the risk of secondary radiation effects. In clinical practice, the implementation of blood vessel‐sparing RT should be carefully optimized to ensure that any increase in MU remains within an acceptable range.

**TABLE 4:** Monitor unit comparison among treatment plans.

	Conv	BVS‐90%	BVS‐80%	Friedman	Post‐hoc *p* values^a^		
	Median (Range) [MU]	Median (Range) [MU]	Median (Range) [MU]	*p* value	Conv vs. BVS‐90%	Conv vs. BVS‐80%	BVS‐90% vs. BVS‐80%
IMRT	1480.1 (1312.8–2153.9)	1458.6 (1304.8–2267.3)	1459.4 (1295.5–2354.1)	0.449	1.000	1.000	1.000
IMPT	2651.5 (2037.0–3393.8)	2639.0 (2102.0–3537.3)	2627.5 (1894.9–3432.5)	0.015	1.000	0.072	0.032

Abbreviations: BVS‐90%, blood vessel sparing plan with 90% dose constraint; BVS‐80%, blood vessel sparing plan with 80% dose constraint; Conv, conventional plan; IMPT, intensity‐modulated proton therapy; IMRT, intensity‐modulated radiation therapy; MU, monitor unit.

^a^ Wilcoxon signed‐rank test with Bonferroni correction.

The cohort included one hypopharyngeal case in which the planning target volumes extensively overlapped with major blood vessels, resulting in a circulating blood dose approximately tenfold higher than that of the oropharyngeal cases. In this case, BVS constraints produced negligible blood dose reduction, as the target volumes and vessels were geometrically inseparable. This observation suggests the dependence of achievable blood dose reduction on the primary tumor site and target‐to‐vessel spatial relationship. By contrast, the remaining 19 cases demonstrated consistent blood dose reductions across both modalities and constraint levels, confirming the general applicability of the BVS approach when sufficient anatomical separation exists between target volumes and major vessels. The efficacy of vessel‐sparing strategies may vary substantially across HNC subtypes, and future studies enrolling larger, disease‐site‐stratified cohorts are warranted to characterize these subtype‐specific differences.

The HEDOS framework provides a systematic approach for calculating radiation dose delivered to circulating blood.^13^, ^15^ Nevertheless, several limitations should be recognized when evaluating radiation‐induced damage to lymphocytes. First, HEDOS‐derived blood dose differs from the actual dose received by all lymphocytes. This is because HEDOS considers only circulating lymphocytes in the blood, excluding stationary lymphocytes located in lymph nodes and other lymphoid organs. Despite this limitation, prior studies have validated circulating blood dose as a clinically relevant surrogate correlated with the occurrence of lymphopenia, a complication known to impair RT outcomes in various types of tumors.^16^, ^17^ According to the study by Kim et al., RT patients with more severe lymphocyte depletion were found to have received higher blood doses as calculated by HEDOS.^16^

Second, HEDOS does not account for patient‐specific tumor vascular alterations. Tumor vasculature is highly irregular and often exposed to substantial radiation. These abnormal hemodynamic properties may introduce discrepancies in circulating blood dose estimates relative to the population‐based models on which HEDOS relies. However, the practical impact of this limitation is likely modest. Blood does not circulate efficiently through tumors, and therefore their contribution to whole‐body blood flow is limited.^30^, ^31^, ^32^ Moreover, tumor vasculature arises from angiogenic sprouting of existing organ vessels, meaning that part of the blood passing through tumor regions is already represented in HEDOS organ‐based circulation modeling.^33^

Given these considerations, HEDOS provides a practical framework through which clinicians can compare relative blood dose across different treatment plans. Incorporating blood dose estimates into the RT plan optimization has potential clinical significance and importance for sparing the immune system.

To assess the uncertainty inherent in automated blood vessel contouring, Dice similarity coefficient was derived from structures contoured using RayStation and TotalSegmentator, respectively (Table SA3). The structures include four major vasculatures included in HEDOS: aorta, large arteries, large veins, and superior vena cava. The large arteries included common carotid arteries, subclavian arteries, and brachiocephalic trunk, while large veins consisted of brachiocephalic veins—all of which could be delineated by both segmentation platforms. The comparative analysis revealed that geometrical complexity of blood vessel governs the robustness of automated segmentation, with morphologically distinct vessels achieving exceptional concordance between the two models. Relatively straightforward structures showed superior Dice similarity coefficients, as exemplified by the aorta (median: 0.917, range: 0.818–0.946) and the superior vena cava (median: 0.841, range: 0.743–0.904). Conversely, more intricate vascular networks demonstrated inferior agreement, with large arteries at 0.641 (range: 0.524–0.696) and large veins at 0.774 (range: 0.393–0.817). To further evaluate whether these geometric discrepancies affect dosimetric outcomes, mean doses to large arteries and large veins were compared between the two segmentation tools across all plans (Table SA4). No statistically significant differences were observed in this study (*p* > 0.05). Nonetheless, all treatment plans should be validated against institutional standards when utilizing automated segmentation tools.

As outlined in Supporting Information Section A1, clinical implementation of blood‐sparing RT will require the development of fast mathematical models that ultimately allow for integrating HEDOS into the optimization loop of RT planning.^11^, ^20^ Such novel optimization approaches would be particularly timely and appropriate, given the increased importance of preserving immune function in modern cancer therapy, and will be developed in future studies. However, even without these advanced implementations, our study demonstrates that blood dose calculation and integration into the planning process are readily achievable using currently available open‐source tools such as TotalSegmentator and HEDOS.

## 5 CONCLUSION

This preliminary planning study investigated the feasibility of quantifying and optimizing the radiation dose delivered to circulating blood of HNC patients depending on treatment modality and dose constraints. In our cohort of 20 HNC patients, IMPT reduced the mean blood dose by 21.5% compared to IMRT. Furthermore, constraining the mean dose delivered to large blood vessels yielded an additional 8%–13% reduction depending on constraint level, without sacrificing tumor target coverage or OAR sparing. Integrating such approaches into RT treatment planning may establish a framework in which immune‐related toxicities are effectively mitigated.

## AUTHOR CONTRIBUTIONS

**Seohan Kim**: Conceptualization; data collection; methodology contribution; manuscript preparation. **Jieun Han**: Data collection; manuscript contribution. **Sebastian Tattenberg**: Conceptualization; data collection; manuscript contribution. **Wonmo Sung**: Conceptualization; methodology contribution; manuscript contribution.

## CONFLICT OF INTEREST STATEMENT

All authors declare no conflicts of interest.

## Supporting information



Supporting Information

## Data Availability

Some research data are stored in an institutional repository and will be shared upon request to the corresponding author.
