# Supplementary material for: Preliminary treatment planning strategy for blood dose reduction in head and neck radiotherapy
Source: J Appl Clin Med Phys. 2026 Apr 13;27(4):e70579. doi: 10.1002/acm2.70579 (PMC13072057; doi:10.1002/acm2.70579)
Supplement: Supplementary file 1 — Supporting Information [file ACM2-27-e70579-s001.docx]

**Supplementary**

**Title: Preliminary treatment planning strategy for blood dose reduction in head and neck radiotherapy**

**
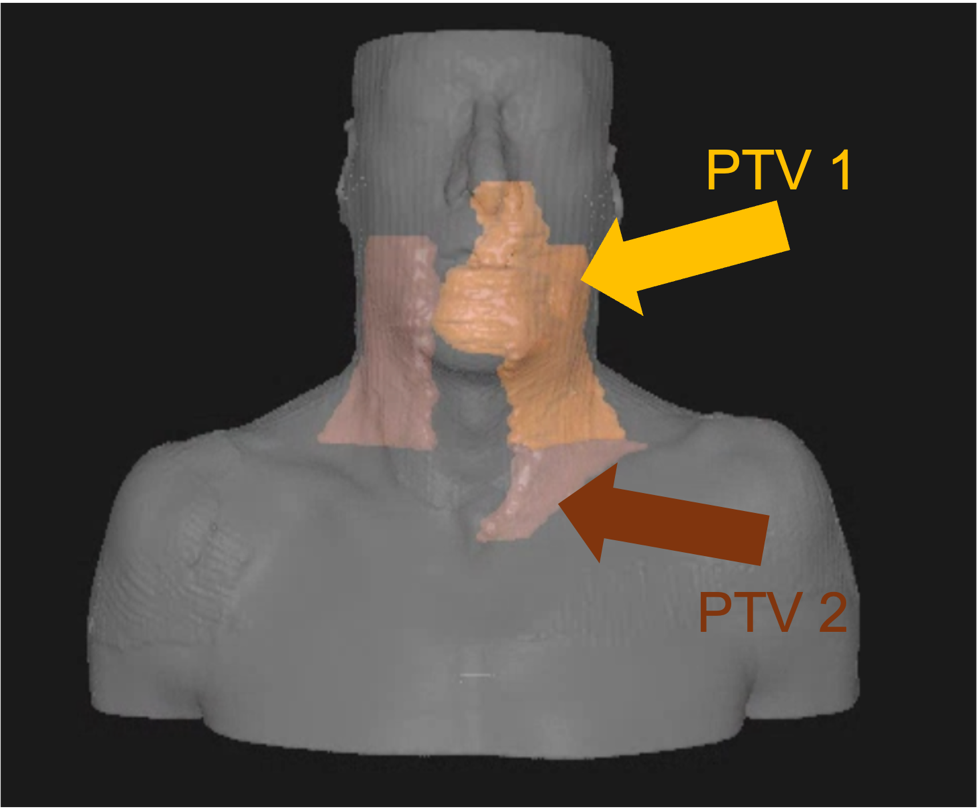
**

Figure A1. The example case of head and neck cancer patient with two planning target volumes (PTVs). PTV1 and PTV2 are for the primary tumor and elective nodal irradiation, respectively.


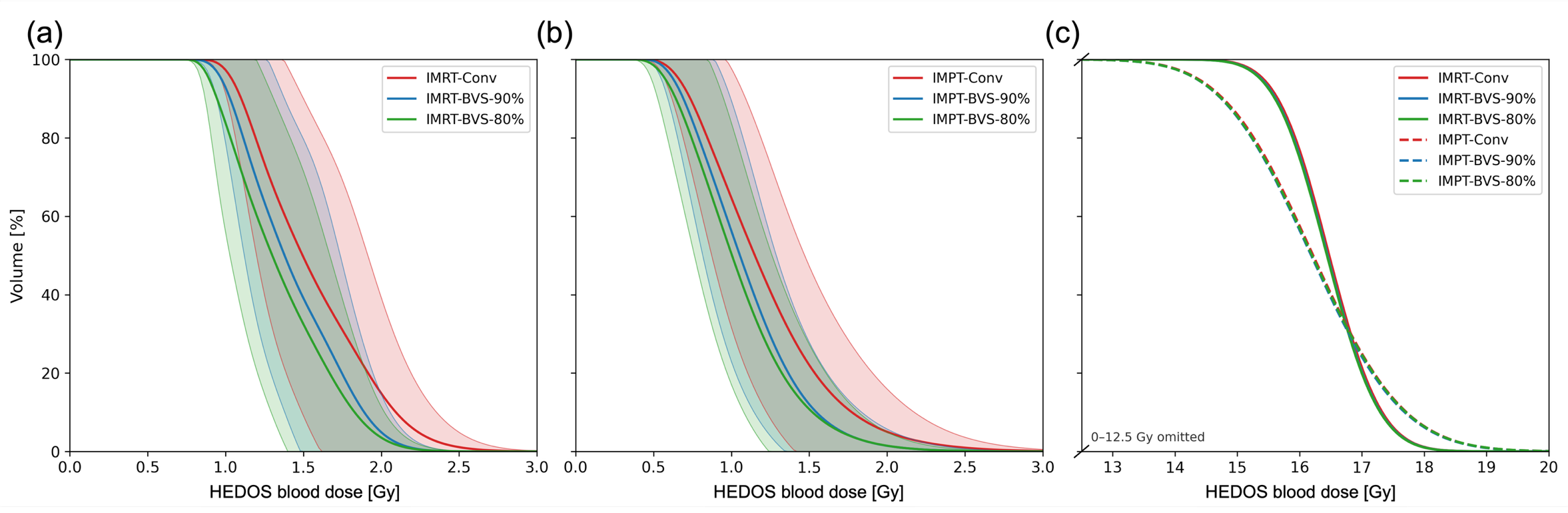


Figure A2. Blood Mean dose-volume histograms (DVHs) of oropharyngeal cases for the comparison of (a) intensity-modulated radiotherapy (IMRT) and (b) intensity-modulated proton therapy (IMPT). Plans include conventional plan (Conv) and blood vessel-sparing (BVS) with 80% or 90% constraints to large vessels. (c) DVHs of hypopharyngeal case includes all plans of IMRT and IMPT.

Table A1-1. Organ-at-risks dose-volume histogram metrics for each treatment plan

| **OAR** | **Metric** | **IMRT** | | | | **IMPT** | | | |
| --- | --- | --- | --- | --- | --- | --- | --- | --- | --- |
|  |  | **Conv** | **BVS-90%** | **BVS-80%** | **p-value** | **Conv** | **BVS-90%** | **BVS-80%** | **p-value** |
| **Brain** | D_max_ | 55.36 (45.89–61.37) | 55.48 (46.56–65.45) | 57.64 (50.22–64.01) | **0.0078** | 53.38 (35.68–60.75) | 54.47 (37.87–62.95) | 54.05 (39.24–61.35) | **0.0474** |
|  | D_mean_ | 3.13 (1.79–8.56) | 3.12 (1.78–8.73) | 3.17 (1.77–8.52) | **0.0172** | 0.97 (0.49–3.02) | 1.20 (0.43–3.12) | 1.02 (0.47–3.09) | 0.0538 |
|  | D_95%_ | 0.14 (0.00–1.07) | 0.15 (0.00–1.08) | 0.13 (0.00–1.04) | **0.0004** | 0.00 (0.00–0.00) | 0.00 (0.00–0.00) | 0.00 (0.00–0.00) | 1.0000 |
|  | D_75%_ | 0.55 (0.18–1.66) | 0.52 (0.18–1.65) | 0.49 (0.17–1.61) | **<0.0001** | 0.00 (0.00–0.01) | 0.00 (0.00–0.01) | 0.00 (0.00–0.01) | <0.0001 |
|  | D_50%_ | 0.95 (0.72–2.94) | 0.93 (0.70–3.00) | 0.89 (0.66–2.86) | **<0.0001** | 0.00 (0.00–0.06) | 0.00 (0.00–0.06) | 0.00 (0.00–0.06) | 0.3679 |
|  | D_25%_ | 2.03 (1.44–10.77) | 2.00 (1.41–11.16) | 1.98 (1.36–10.87) | **<0.0001** | 0.02 (0.00–2.24) | 0.02 (0.00–2.36) | 0.02 (0.00–2.35) | 0.7788 |
|  | D_5%_ | 17.69 (4.46–38.31) | 17.38 (4.50–39.24) | 18.30 (4.38–38.87) | 0.1165 | 6.54 (0.68–20.78) | 9.55 (0.58–21.56) | 6.96 (0.50–18.18) | **0.0278** |
|  | D_2%_ | 34.73 (16.99–46.74) | 34.41 (16.24–47.30) | 33.61 (15.49–46.88) | 0.2466 | 16.68 (8.02–32.69) | 17.73 (7.34–33.78) | 16.96 (6.90–31.33) | 0.3867 |
| **Brainstem** | D_max_ | 51.07 (43.66–54.92) | 51.75 (44.76–55.19) | 52.69 (45.00–55.61) | **0.0106** | 41.02 (25.01–55.13) | 43.43 (27.64–54.63) | 45.02 (30.03–52.64) | **0.0474** |
|  | D_mean_ | 10.16 (4.55–21.96) | 10.12 (4.51–22.44) | 10.15 (4.48–21.05) | 0.9512 | 5.12 (1.13–12.03) | 5.40 (1.04–12.44) | 5.46 (1.13–11.90) | **0.0111** |
|  | D_95%_ | 1.77 (1.36–3.36) | 1.77 (1.34–3.30) | 1.74 (1.30–3.08) | **<0.0001** | 0.01 (0.00–0.09) | 0.01 (0.00–0.11) | 0.01 (0.00–0.09) | 0.1496 |
|  | D_75%_ | 2.55 (1.86–5.94) | 2.50 (1.82–6.01) | 2.46 (1.74–4.64) | **<0.0001** | 0.06 (0.02–0.42) | 0.06 (0.02–0.93) | 0.05 (0.02–0.44) | 0.9246 |
|  | D_50%_ | 3.75 (2.66–18.96) | 3.65 (2.56–19.16) | 3.63 (2.48–18.94) | **0.0039** | 0.26 (0.06–6.26) | 0.31 (0.06–7.54) | 0.26 (0.06–6.88) | 0.0833 |
|  | D_25%_ | 9.76 (3.75–38.64) | 9.44 (3.65–39.53) | 9.76 (3.52–36.71) | 0.1653 | 4.01 (0.21–23.81) | 5.17 (0.19–24.74) | 4.04 (0.20–23.29) | 0.0853 |
|  | D_5%_ | 43.13 (17.83–49.51) | 42.98 (17.90–50.04) | 44.61 (17.34–51.48) | 0.0863 | 28.38 (8.09–40.60) | 27.55 (7.30–41.76) | 28.83 (7.85–41.85) | **0.0429** |
|  | D_2%_ | 45.55 (26.38–50.81) | 45.52 (26.79–52.17) | 47.11 (27.34–52.73) | **0.0013** | 31.66 (17.04–45.18) | 33.33 (15.08–45.82) | 32.77 (16.60–43.87) | 0.0743 |
| **Esophagus** | D_max_ | 52.65 (46.81–63.53) | 52.79 (47.64–65.83) | 52.67 (45.00–74.77) | 0.7047 | 57.34 (49.46–67.54) | 57.28 (52.15–67.08) | 57.57 (48.92–67.75) | 1.0000 |
|  | D_mean_ | 27.99 (10.14–39.87) | 26.11 (9.06–38.08) | 24.07 (7.41–35.36) | **<0.0001** | 24.25 (7.61–40.43) | 22.32 (6.71–39.04) | 20.26 (5.77–37.99) | **<0.0001** |
|  | D_95%_ | 2.12 (0.04–22.82) | 1.92 (0.03–19.89) | 1.68 (0.03–19.51) | **<0.0001** | 0.18 (0.00–10.55) | 0.12 (0.00–10.43) | 0.10 (0.00–10.02) | **<0.0001** |
|  | D_75%_ | 3.92 (0.60–38.00) | 3.56 (0.48–36.73) | 3.26 (0.42–33.33) | **<0.0001** | 1.19 (0.00–35.17) | 0.50 (0.00–30.41) | 0.33 (0.00–26.10) | **<0.0001** |
|  | D_50%_ | 30.52 (1.89–48.52) | 26.67 (1.72–45.55) | 24.52 (1.64–43.20) | **<0.0001** | 26.51 (0.10–47.89) | 17.27 (0.07–46.88) | 8.28 (0.05–47.61) | **<0.0001** |
|  | D_25%_ | 45.37 (19.90–50.77) | 43.91 (16.38–50.49) | 42.09 (11.32–49.05) | **<0.0001** | 47.67 (12.23–53.48) | 47.01 (4.26–53.15) | 44.86 (1.42–53.15) | **0.0016** |
|  | D_5%_ | 50.91 (36.62–56.61) | 50.21 (34.81–57.84) | 49.48 (31.74–65.57) | **0.0010** | 53.45 (30.69–63.44) | 53.69 (33.67–63.16) | 54.45 (26.83–62.96) | 0.1270 |
|  | D_2%_ | 51.78 (38.08–60.76) | 51.66 (37.26–62.03) | 50.95 (36.54–69.41) | **0.0007** | 54.96 (33.64–65.47) | 54.52 (36.23–64.89) | 55.70 (29.94–64.93) | 0.3867 |
| **Larynx** | D_max_ | 60.98 (60.35–63.17) | 61.02 (60.16–62.59) | 61.05 (60.12–63.01) | 0.8607 | 61.98 (60.74–63.52) | 61.97 (60.79–63.42) | 61.85 (60.87–63.98) | 0.9512 |
|  | D_mean_ | 43.42 (41.83–46.56) | 43.31 (42.44–46.52) | 42.97 (41.83–46.63) | **0.0023** | 44.03 (43.92–47.77) | 44.01 (40.74–47.57) | 44.05 (43.72–47.87) | 0.1578 |
|  | D_95%_ | 32.95 (20.52–35.91) | 32.48 (20.44–35.83) | 31.66 (20.75–35.01) | **0.0004** | 29.84 (14.99–33.66) | 29.59 (14.42–33.14) | 29.22 (14.38–33.31) | 0.7047 |
|  | D_75%_ | 36.83 (30.09–38.99) | 36.69 (30.36–38.38) | 36.11 (30.37–38.24) | **0.0058** | 37.42 (31.91–40.18) | 37.05 (29.61–39.62) | 37.35 (33.26–39.97) | **0.0429** |
|  | D_50%_ | 41.99 (35.50–56.63) | 41.86 (37.77–56.73) | 41.34 (38.97–56.74) | 0.1165 | 44.16 (42.93–56.94) | 43.94 (42.51–56.85) | 44.28 (43.00–57.25) | 0.0784 |
|  | D_25%_ | 48.75 (47.35–58.94) | 48.63 (47.25–58.98) | 48.48 (46.91–59.03) | 0.7047 | 51.12 (48.65–58.65) | 51.36 (48.99–58.58) | 50.97 (49.24–58.76) | **0.0316** |
|  | D_5%_ | 58.91 (55.92–60.70) | 58.73 (55.21–60.28) | 58.57 (55.65–60.70) | 0.4620 | 58.30 (55.88–60.58) | 58.84 (56.34–60.56) | 58.50 (56.39–60.57) | 0.1857 |
|  | D_2%_ | 59.99 (57.99–61.32) | 59.83 (57.55–60.62) | 59.78 (58.38–61.00) | 0.2592 | 59.62 (57.68–61.14) | 59.80 (58.11–61.04) | 59.54 (58.00–61.88) | 0.0785 |
| **Mandible** | D_max_ | 68.87 (61.87–77.37) | 68.72 (61.18–78.93) | 69.19 (62.41–80.47) | **0.0106** | 62.20 (55.27–64.39) | 62.10 (54.22–65.25) | 61.95 (54.68–63.82) | 0.1572 |
|  | D_mean_ | 40.45 (36.47–46.38) | 40.60 (36.39–46.46) | 41.06 (36.81–46.64) | 0.3499 | 27.92 (22.12–37.04) | 28.29 (23.76–36.59) | 28.00 (23.38–37.63) | 0.1148 |
|  | D_95%_ | 15.51 (7.68–24.16) | 15.18 (6.90–23.14) | 13.89 (6.68–23.50) | **0.0003** | 4.65 (1.22–12.01) | 4.55 (1.44–18.56) | 4.42 (1.59–13.21) | 0.1572 |
|  | D_75%_ | 31.14 (26.49–39.43) | 31.73 (26.12–39.37) | 31.80 (27.82–39.85) | 0.5220 | 17.00 (11.97–22.87) | 18.02 (12.52–28.50) | 17.37 (12.20–25.01) | **0.0429** |
|  | D_50%_ | 40.01 (36.50–48.81) | 40.60 (36.47–48.14) | 41.01 (36.71–50.57) | 0.2592 | 23.75 (16.57–36.72) | 24.99 (16.69–36.28) | 23.86 (17.27–38.09) | 0.0907 |
|  | D_25%_ | 51.17 (41.97–61.02) | 50.86 (41.78–60.57) | 50.70 (42.88–60.86) | 0.1148 | 40.83 (29.71–53.28) | 41.74 (32.37–52.98) | 40.88 (30.79–53.50) | 0.2004 |
|  | D_5%_ | 61.39 (50.25–69.87) | 61.37 (50.48–69.11) | 61.73 (50.89–69.93) | 0.3499 | 58.57 (49.00–60.94) | 58.64 (49.09–60.21) | 58.59 (49.30–60.12) | 0.1519 |
|  | D_2%_ | 63.85 (51.99–71.74) | 63.95 (52.42–74.29) | 64.39 (52.98–75.25) | 0.1572 | 60.22 (51.03–61.94) | 59.99 (51.20–60.95) | 60.12 (51.54–60.94) | **0.0096** |
| **Oral Cavity** | D_max_ | 65.56 (61.59–76.10) | 65.58 (61.55–75.13) | 66.35 (61.44–77.19) | 0.5220 | 62.60 (56.67–67.46) | 62.60 (58.18–67.87) | 62.45 (58.04–67.46) | 0.7105 |
|  | D_mean_ | 42.71 (31.75–55.46) | 43.39 (31.63–55.10) | 42.67 (31.77–55.32) | 0.1423 | 27.06 (13.92–52.65) | 28.94 (14.52–52.88) | 27.28 (14.46–53.34) | **0.0351** |
|  | D_95%_ | 25.08 (0.00–35.35) | 25.27 (0.00–35.47) | 25.62 (0.00–35.60) | **0.0472** | 1.01 (0.00–21.31) | 1.01 (0.00–20.79) | 0.94 (0.00–21.29) | 0.4381 |
|  | D_75%_ | 34.12 (24.10–54.17) | 34.23 (23.97–53.26) | 34.07 (24.21–53.28) | 0.8465 | 14.56 (4.27–52.26) | 14.93 (4.61–52.98) | 14.90 (4.50–54.19) | **0.0429** |
|  | D_50%_ | 42.52 (30.53–59.74) | 42.75 (30.62–59.59) | 42.12 (30.65–59.64) | 0.9874 | 22.98 (15.85–59.40) | 24.79 (16.49–59.61) | 23.08 (16.68–59.42) | **0.0377** |
|  | D_25%_ | 51.36 (37.80–60.20) | 51.02 (37.93–60.17) | 50.53 (37.88–60.17) | 0.8187 | 41.74 (19.35–60.19) | 44.30 (20.07–60.20) | 41.21 (19.97–60.23) | 0.0810 |
|  | D_5%_ | 60.45 (49.58–61.48) | 60.05 (50.25–61.92) | 60.10 (50.36–61.23) | 0.7047 | 58.42 (30.11–61.13) | 58.31 (31.57–60.87) | 58.69 (31.03–61.26) | 0.8607 |
|  | D_2%_ | 60.96 (53.06–65.82) | 60.75 (53.86–65.83) | 61.02 (53.49–65.76) | 0.5378 | 60.53 (37.82–61.61) | 60.41 (39.40–61.15) | 60.74 (39.03–61.81) | 0.9142 |
| **Optic Chiasm** | D_max_ | 2.52 (1.46–3.65) | 2.48 (1.37–3.54) | 2.46 (1.30–3.56) | **<0.0001** | 0.13 (0.02–0.40) | 0.12 (0.02–0.38) | 0.13 (0.04–0.42) | 0.6422 |
|  | D_mean_ | 2.30 (1.38–3.39) | 2.25 (1.31–3.29) | 2.23 (1.25–3.31) | **<0.0001** | 0.07 (0.01–0.24) | 0.07 (0.01–0.22) | 0.07 (0.01–0.24) | 0.4056 |
|  | D_95%_ | 2.02 (1.21–3.13) | 1.98 (1.20–3.02) | 1.94 (1.16–3.04) | **<0.0001** | 0.03 (0.00–0.13) | 0.03 (0.00–0.13) | 0.02 (0.00–0.14) | 0.8498 |
|  | D_75%_ | 2.14 (1.31–3.18) | 2.05 (1.24–3.10) | 2.04 (1.20–3.12) | **<0.0001** | 0.04 (0.00–0.17) | 0.04 (0.00–0.16) | 0.04 (0.00–0.20) | 0.2847 |
|  | D_50%_ | 2.21 (1.35–3.56) | 2.17 (1.34–3.42) | 2.15 (1.28–3.42) | **<0.0001** | 0.07 (0.01–0.24) | 0.07 (0.00–0.22) | 0.06 (0.01–0.24) | 0.4626 |
|  | D_25%_ | 2.47 (1.43–3.61) | 2.44 (1.35–3.51) | 2.41 (1.29–3.53) | **<0.0001** | 0.09 (0.01–0.28) | 0.09 (0.01–0.25) | 0.09 (0.02–0.31) | 0.9112 |
|  | D_5%_ | 2.51 (1.44–3.65) | 2.46 (1.36–3.54) | 2.45 (1.30–3.56) | **<0.0001** | 0.12 (0.02–0.35) | 0.12 (0.02–0.38) | 0.11 (0.03–0.36) | 0.8964 |
|  | D_2%_ | 2.51 (1.44–3.65) | 2.48 (1.36–3.54) | 2.46 (1.30–3.56) | **<0.0001** | 0.12 (0.02–0.40) | 0.12 (0.02–0.38) | 0.11 (0.04–0.36) | 0.6016 |
| **Optic Nerve L** | D_max_ | 2.46 (1.60–4.13) | 2.40 (1.59–4.02) | 2.37 (1.53–4.08) | **<0.0001** | 0.12 (0.03–0.40) | 0.11 (0.03–0.47) | 0.11 (0.04–0.44) | 0.1765 |
|  | D_mean_ | 1.86 (1.31–3.55) | 1.87 (1.32–3.44) | 1.85 (1.27–3.47) | **<0.0001** | 0.03 (0.01–0.18) | 0.03 (0.01–0.18) | 0.03 (0.01–0.16) | 0.9733 |
|  | D_95%_ | 1.50 (0.84–3.07) | 1.50 (0.78–2.98) | 1.47 (0.69–2.99) | **<0.0001** | 0.00 (0.00–0.06) | 0.00 (0.00–0.05) | 0.00 (0.00–0.07) | **0.0147** |
|  | D_75%_ | 1.65 (1.14–3.36) | 1.60 (1.16–3.26) | 1.58 (1.13–3.27) | **<0.0001** | 0.01 (0.00–0.10) | 0.01 (0.00–0.12) | 0.01 (0.00–0.11) | 0.5021 |
|  | D_50%_ | 1.90 (1.30–3.46) | 1.84 (1.31–3.35) | 1.81 (1.28–3.36) | **<0.0001** | 0.02 (0.00–0.13) | 0.02 (0.00–0.13) | 0.02 (0.00–0.14) | 0.1095 |
|  | D_25%_ | 2.05 (1.45–3.86) | 2.02 (1.42–3.74) | 2.01 (1.38–3.80) | **<0.0001** | 0.04 (0.01–0.28) | 0.04 (0.01–0.28) | 0.04 (0.01–0.24) | 0.7985 |
|  | D_5%_ | 2.33 (1.56–3.96) | 2.22 (1.51–3.83) | 2.20 (1.46–3.89) | **<0.0001** | 0.08 (0.02–0.33) | 0.08 (0.02–0.35) | 0.08 (0.03–0.27) | 0.1744 |
|  | D_2%_ | 2.38 (1.59–3.96) | 2.29 (1.56–3.83) | 2.26 (1.51–3.89) | **<0.0001** | 0.09 (0.03–0.36) | 0.08 (0.02–0.35) | 0.09 (0.03–0.27) | 0.3679 |
| **Optic Nerve R** | D_max_ | 2.42 (1.56–3.87) | 2.42 (1.57–3.86) | 2.36 (1.52–3.90) | **<0.0001** | 0.09 (0.03–0.37) | 0.09 (0.03–0.45) | 0.08 (0.02–0.52) | 0.3955 |
|  | D_mean_ | 1.92 (1.32–3.20) | 1.86 (1.31–3.14) | 1.80 (1.29–3.17) | **<0.0001** | 0.02 (0.01–0.14) | 0.02 (0.01–0.15) | 0.02 (0.01–0.15) | 0.6246 |
|  | D_95%_ | 1.56 (0.93–2.48) | 1.52 (0.90–2.44) | 1.46 (0.87–2.46) | **<0.0001** | 0.00 (0.00–0.03) | 0.00 (0.00–0.03) | 0.00 (0.00–0.03) | **0.0183** |
|  | D_75%_ | 1.69 (1.24–2.90) | 1.67 (1.23–2.85) | 1.61 (1.21–2.88) | **<0.0001** | 0.00 (0.00–0.06) | 0.01 (0.00–0.07) | 0.00 (0.00–0.06) | 0.3583 |
|  | D_50%_ | 1.94 (1.31–3.23) | 1.86 (1.30–3.17) | 1.80 (1.29–3.20) | **<0.0001** | 0.01 (0.00–0.12) | 0.02 (0.00–0.13) | 0.02 (0.00–0.15) | 0.1750 |
|  | D_25%_ | 2.08 (1.38–3.39) | 2.06 (1.38–3.33) | 2.02 (1.33–3.37) | **<0.0001** | 0.04 (0.01–0.21) | 0.04 (0.01–0.27) | 0.03 (0.01–0.24) | 0.5875 |
|  | D_5%_ | 2.29 (1.46–3.71) | 2.26 (1.45–3.64) | 2.24 (1.44–3.66) | **0.0003** | 0.06 (0.03–0.31) | 0.06 (0.02–0.37) | 0.06 (0.02–0.30) | 0.2096 |
|  | D_2%_ | 2.35 (1.46–3.76) | 2.31 (1.45–3.66) | 2.29 (1.44–3.68) | **<0.0001** | 0.07 (0.03–0.34) | 0.08 (0.02–0.40) | 0.08 (0.02–0.38) | 0.9412 |
| **Parotid L** | D_max_ | 61.00 (52.33–62.26) | 60.96 (52.39–62.40) | 61.03 (52.73–64.00) | **0.0037** | 62.06 (53.18–64.48) | 61.78 (52.93–64.78) | 62.25 (53.39–65.26) | 0.8607 |
|  | D_mean_ | 20.09 (18.14–24.23) | 20.13 (18.03–24.26) | 20.09 (17.85–24.51) | **0.0421** | 20.05 (16.52–24.70) | 20.08 (16.29–24.75) | 20.05 (16.30–24.75) | 0.8483 |
|  | D_95%_ | 8.79 (7.02–13.77) | 8.61 (6.72–13.14) | 8.30 (6.76–12.85) | **0.0004** | 0.61 (0.15–5.69) | 0.52 (0.07–5.49) | 0.49 (0.07–5.40) | 0.0597 |
|  | D_75%_ | 10.89 (8.96–14.72) | 10.64 (9.05–14.27) | 10.19 (8.78–13.84) | **0.0020** | 3.46 (1.44–7.67) | 3.42 (1.06–7.03) | 3.40 (0.97–7.58) | **0.0479** |
|  | D_50%_ | 13.31 (11.55–16.90) | 13.09 (11.28–17.59) | 12.88 (11.11–17.02) | **0.0293** | 12.47 (7.59–18.57) | 12.14 (5.91–18.45) | 11.82 (5.90–18.37) | 0.1556 |
|  | D_25%_ | 24.25 (19.18–36.57) | 24.17 (18.11–37.32) | 24.79 (16.91–37.99) | 0.4505 | 35.89 (28.54–45.53) | 36.57 (28.95–45.53) | 35.86 (29.26–46.21) | 0.2466 |
|  | D_5%_ | 53.97 (39.58–59.79) | 54.78 (40.26–59.68) | 55.51 (40.80–59.74) | **0.0013** | 56.45 (48.10–60.46) | 57.24 (47.84–60.23) | 57.16 (47.57–60.29) | 0.2466 |
|  | D_2%_ | 59.23 (45.16–60.38) | 58.91 (47.19–60.39) | 58.48 (48.48–60.26) | 0.2366 | 59.33 (50.66–61.32) | 59.38 (51.37–61.27) | 59.51 (50.79–61.69) | 0.2929 |
| **Parotid R** | D_max_ | 61.08 (52.22–62.51) | 61.13 (52.52–63.27) | 61.23 (52.58–63.21) | 0.1479 | 62.30 (53.08–64.69) | 61.52 (53.02–65.26) | 61.77 (53.44–64.20) | 0.1572 |
|  | D_mean_ | 20.09 (17.91–24.03) | 20.12 (18.04–24.23) | 20.09 (18.03–24.46) | **0.0060** | 20.02 (17.04–25.05) | 20.03 (16.76–24.91) | 20.02 (16.84–25.16) | 0.2335 |
|  | D_95%_ | 8.84 (5.87–11.14) | 9.00 (5.96–10.89) | 8.52 (5.84–11.03) | **0.0104** | 0.43 (0.03–8.10) | 0.23 (0.03–6.64) | 0.42 (0.03–6.89) | **0.0005** |
|  | D_75%_ | 10.98 (9.12–12.92) | 10.88 (9.12–12.88) | 10.36 (8.31–12.94) | **0.0260** | 2.85 (1.55–10.48) | 2.51 (1.20–9.03) | 2.79 (1.09–9.21) | **0.0224** |
|  | D_50%_ | 13.25 (11.49–15.94) | 13.25 (11.56–15.93) | 12.88 (10.72–16.07) | **0.0031** | 12.11 (7.62–18.27) | 12.04 (7.26–17.76) | 12.41 (7.17–17.80) | **0.0174** |
|  | D_25%_ | 24.04 (17.75–34.62) | 23.68 (16.90–36.57) | 24.50 (18.08–39.46) | **0.0023** | 32.89 (28.34–47.41) | 34.61 (28.68–46.32) | 33.20 (28.72–48.70) | 0.1165 |
|  | D_5%_ | 53.39 (43.47–60.13) | 54.94 (43.33–59.88) | 56.04 (44.71–59.22) | **0.0193** | 56.73 (44.33–61.14) | 57.45 (47.78–60.67) | 57.10 (47.54–61.01) | **0.0144** |
|  | D_2%_ | 58.67 (48.71–60.56) | 59.00 (48.56–60.45) | 58.92 (49.16–60.30) | 0.4493 | 59.47 (47.83–61.82) | 59.45 (50.66–61.21) | 59.57 (50.30–61.60) | 0.1572 |
| **Spinal Cord** | D_max_ | 53.16 (50.04–60.60) | 53.31 (50.17–59.73) | 52.93 (50.38–60.40) | 0.2865 | 49.08 (36.77–61.05) | 50.77 (37.57–60.50) | 50.85 (37.38–59.38) | **0.0118** |
|  | D_mean_ | 31.73 (20.77–43.74) | 31.12 (20.53–44.18) | 29.88 (20.26–43.89) | **<0.0001** | 19.93 (12.02–30.32) | 19.73 (12.10–29.50) | 19.68 (10.70–28.94) | **0.0045** |
|  | D_95%_ | 1.71 (0.02–34.34) | 1.50 (0.01–34.36) | 1.42 (0.01–32.20) | **<0.0001** | 0.02 (0.00–21.26) | 0.01 (0.00–13.30) | 0.01 (0.00–8.94) | **0.0013** |
|  | D_75%_ | 10.59 (0.51–42.79) | 9.84 (0.46–41.96) | 9.10 (0.41–41.07) | **0.0028** | 2.13 (0.00–23.66) | 1.97 (0.00–23.11) | 1.97 (0.00–21.82) | **<0.0001** |
|  | D_50%_ | 43.04 (19.62–46.45) | 42.57 (17.94–46.51) | 40.79 (13.18–46.20) | **<0.0001** | 22.45 (5.67–31.46) | 22.73 (2.09–31.88) | 22.91 (0.98–32.13) | 0.7047 |
|  | D_25%_ | 46.44 (42.06–47.94) | 46.43 (41.66–48.01) | 46.04 (41.58–47.99) | **0.0221** | 27.57 (20.36–40.36) | 27.93 (19.26–40.12) | 28.85 (17.24–41.15) | 0.1572 |
|  | D_5%_ | 49.00 (47.49–50.14) | 49.02 (47.67–50.54) | 48.72 (47.75–50.92) | 0.2472 | 36.38 (29.99–45.95) | 37.33 (24.34–46.22) | 38.05 (25.98–46.54) | 0.2122 |
|  | D_2%_ | 49.90 (48.51–51.16) | 49.96 (48.76–51.64) | 49.83 (48.66–52.15) | 0.3160 | 40.14 (32.92–49.16) | 41.73 (28.25–49.68) | 41.73 (32.72–49.68) | 0.1998 |
| **Large Arteries** | D_max_ | 61.66 (60.66–63.67) | 61.80 (60.34–64.33) | 61.45 (59.97–64.65) | 0.3499 | 62.22 (61.06–63.35) | 61.93 (60.80–62.98) | 61.89 (60.54–62.97) | **0.0429** |
|  | D_mean_ | 34.05 (18.52–54.09) | 30.70 (16.61–53.67) | 27.92 (14.79–50.96) | **<0.0001** | 32.77 (12.50–53.48) | 29.59 (11.22–50.28) | 26.91 (10.03–48.87) | **<0.0001** |
|  | D_95%_ | 2.43 (0.62–49.00) | 1.94 (0.55–44.45) | 1.68 (0.54–33.62) | **<0.0001** | 0.08 (0.00–43.00) | 0.07 (0.00–20.25) | 0.05 (0.00–10.36) | **<0.0001** |
|  | D_75%_ | 6.97 (2.72–51.72) | 5.31 (2.34–51.20) | 4.45 (2.04–49.72) | **<0.0001** | 2.21 (0.09–51.36) | 0.98 (0.08–50.12) | 0.60 (0.05–49.47) | **<0.0001** |
|  | D_50%_ | 49.37 (10.97–52.20) | 32.96 (7.72–52.08) | 19.64 (6.06–51.70) | **<0.0001** | 47.72 (0.67–52.71) | 36.60 (0.46–52.00) | 25.04 (0.34–51.26) | **<0.0001** |
|  | D_25%_ | 52.16 (30.18–59.90) | 51.89 (23.69–58.96) | 51.28 (16.98–57.41) | **<0.0001** | 52.28 (17.76–59.48) | 51.99 (10.58–59.16) | 51.64 (5.91–59.12) | **<0.0001** |
|  | D_5%_ | 60.31 (52.28–60.68) | 59.66 (52.21–60.64) | 58.97 (52.30–60.30) | **<0.0001** | 60.18 (52.73–60.78) | 59.84 (52.39–60.32) | 59.51 (52.95–60.11) | **0.0002** |
|  | D_2%_ | 60.54 (59.88–61.03) | 59.91 (59.22–61.02) | 59.50 (58.74–60.65) | **<0.0001** | 60.79 (59.68–61.28) | 60.36 (59.12–60.79) | 60.12 (59.48–60.78) | **<0.0001** |
| **Large Veins** | D_max_ | 61.88 (60.66–63.03) | 61.60 (60.21–63.98) | 61.28 (60.13–63.05) | **0.0006** | 62.70 (61.20–66.09) | 62.32 (60.99–65.74) | 62.04 (60.44–65.26) | **0.0408** |
|  | D_mean_ | 26.21 (16.26–52.20) | 23.62 (14.70–48.60) | 21.77 (13.12–46.19) | **<0.0001** | 25.77 (12.28–52.48) | 23.29 (11.09–49.80) | 21.35 (8.30–49.35) | **<0.0001** |
|  | D_95%_ | 1.62 (0.66–49.86) | 1.44 (0.56–31.60) | 1.24 (0.47–27.80) | **<0.0001** | 0.05 (0.00–50.30) | 0.04 (0.00–40.33) | 0.04 (0.00–41.56) | **<0.0001** |
|  | D_75%_ | 3.34 (1.59–51.42) | 2.79 (1.35–49.36) | 2.49 (1.19–46.00) | **<0.0001** | 0.54 (0.05–51.41) | 0.38 (0.05–49.92) | 0.30 (0.03–49.49) | **<0.0001** |
|  | D_50%_ | 11.80 (3.18–51.99) | 7.94 (2.67–51.40) | 6.63 (2.33–51.48) | **<0.0001** | 15.20 (0.26–52.09) | 5.55 (0.21–51.06) | 2.33 (0.18–50.22) | **<0.0001** |
|  | D_25%_ | 52.13 (21.36–59.51) | 51.39 (11.47–55.84) | 50.41 (9.54–54.48) | **<0.0001** | 52.19 (17.51–59.10) | 51.77 (10.79–58.57) | 51.27 (2.11–52.67) | **<0.0001** |
|  | D_5%_ | 60.24 (52.22–60.47) | 59.39 (52.02–60.28) | 58.44 (51.33–60.27) | **<0.0001** | 60.28 (52.43–60.92) | 59.86 (52.09–60.31) | 59.19 (52.41–59.93) | **<0.0001** |
|  | D_2%_ | 60.62 (59.64–60.89) | 59.81 (59.07–60.82) | 59.02 (58.16–60.60) | **<0.0001** | 60.88 (59.36–61.44) | 60.34 (58.67–60.97) | 59.75 (58.91–60.45) | **<0.0001** |

Friedman test with post-hoc Wilcoxon signed-rank tests (Bonferroni-corrected, n=20). Values are median (range) in Gy. Bold p-values indicate p < 0.05.

Abbreviations: BVS, blood vessel-sparing plan; Conv, conventional plan; D_max_, maximum dose; D_mean_, mean dose; D_n%_, The minimum dose received by at least n% of the blood volume; IMRT, intensity-modulated radiation therapy; IMPT, intensity-modulated proton therapy; OAR, organ-at-risk.

Table A1-2. Post-hoc analysis of organ-at-risks dose-volume histogram metrics (significant only)

| **OAR** | **Modality** | **Metric** | **Conv vs BVS-90%** | **Conv vs BVS-80%** | **BVS-90% vs BVS-80%** |
| --- | --- | --- | --- | --- | --- |
| Brain | IMRT | Dmax | 0.1087 | **0.0014** | **0.0459** |
| Brain | IMRT | Dmean | 0.2296 | 0.1200 | 0.4159 |
| Brain | IMRT | D95% | 0.2466 | **0.0065** | **0.0325** |
| Brain | IMRT | D75% | **0.0006** | **<0.0001** | **0.0006** |
| Brain | IMRT | D50% | **0.0173** | **0.0004** | **0.0018** |
| Brain | IMRT | D25% | **0.0146** | **0.0008** | **0.0156** |
| Brain | IMPT | Dmax | **0.0026** | 0.0515 | 1.0000 |
| Brain | IMPT | D5% | 0.1200 | 1.0000 | **0.0356** |
| Brainstem | IMRT | Dmax | 0.3420 | **0.0021** | **0.0321** |
| Brainstem | IMRT | D95% | **0.0016** | **<0.0001** | **0.0006** |
| Brainstem | IMRT | D75% | **0.0071** | **<0.0001** | **0.0010** |
| Brainstem | IMRT | D50% | 0.2691 | **0.0219** | 0.4287 |
| Brainstem | IMRT | D2% | **0.0167** | **0.0002** | 0.0645 |
| Brainstem | IMPT | Dmax | **0.0043** | 0.2919 | 1.0000 |
| Brainstem | IMPT | Dmean | **0.0051** | 0.1757 | 1.0000 |
| Brainstem | IMPT | D5% | **0.0127** | 0.1595 | 1.0000 |
| Esophagus | IMRT | Dmean | **<0.0001** | **<0.0001** | **0.0001** |
| Esophagus | IMRT | D95% | **<0.0001** | **<0.0001** | **0.0004** |
| Esophagus | IMRT | D75% | **<0.0001** | **<0.0001** | **<0.0001** |
| Esophagus | IMRT | D50% | **<0.0001** | **<0.0001** | **0.0001** |
| Esophagus | IMRT | D25% | **0.0014** | **0.0002** | **0.0006** |
| Esophagus | IMRT | D5% | **0.0036** | **0.0110** | 0.1200 |
| Esophagus | IMRT | D2% | **0.0095** | 0.1087 | 0.3693 |
| Esophagus | IMPT | Dmean | **<0.0001** | **<0.0001** | **0.0043** |
| Esophagus | IMPT | D95% | **0.0029** | **0.0029** | **0.0029** |
| Esophagus | IMPT | D75% | **0.0014** | **0.0041** | **0.0184** |
| Esophagus | IMPT | D50% | **<0.0001** | **0.0005** | 0.0799 |
| Esophagus | IMPT | D25% | **0.0043** | **0.0012** | **0.0067** |
| Larynx | IMRT | Dmean | 0.1250 | **0.0051** | 0.0799 |
| Larynx | IMRT | D95% | 0.2276 | **0.0030** | **0.0283** |
| Larynx | IMRT | D75% | 0.2276 | **0.0081** | **0.0408** |
| Larynx | IMPT | D75% | 0.0577 | 0.3420 | 0.8321 |
| Larynx | IMPT | D25% | 1.0000 | 0.6818 | 0.1322 |
| Mandible | IMRT | Dmax | 0.7833 | 0.2691 | **0.0219** |
| Mandible | IMRT | D95% | **0.0014** | **0.0051** | 0.2199 |
| Mandible | IMPT | D75% | 0.2087 | 0.1200 | 1.0000 |
| Mandible | IMPT | D2% | **0.0408** | 1.0000 | **0.0167** |
| Oral Cavity | IMRT | D95% | 0.0933 | **0.0186** | 0.3496 |
| Oral Cavity | IMPT | Dmean | 0.0515 | 0.3693 | 0.9354 |
| Oral Cavity | IMPT | D75% | 0.4287 | 0.1595 | 1.0000 |
| Oral Cavity | IMPT | D50% | **0.0223** | 0.2477 | 0.2919 |
| Optic Chiasm | IMRT | Dmax | **0.0127** | **0.0058** | **0.0091** |
| Optic Chiasm | IMRT | Dmean | **0.0030** | **<0.0001** | **0.0010** |
| Optic Chiasm | IMRT | D95% | **0.0012** | **0.0004** | **<0.0001** |
| Optic Chiasm | IMRT | D75% | **0.0167** | **0.0001** | **0.0025** |
| Optic Chiasm | IMRT | D50% | **0.0030** | **0.0004** | **0.0005** |
| Optic Chiasm | IMRT | D25% | **0.0037** | **<0.0001** | **0.0010** |
| Optic Chiasm | IMRT | D5% | **0.0080** | **0.0006** | **0.0084** |
| Optic Chiasm | IMRT | D2% | **0.0124** | **0.0021** | **0.0127** |
| Optic Nerve L | IMRT | Dmax | **0.0014** | **0.0004** | **0.0066** |
| Optic Nerve L | IMRT | Dmean | **0.0004** | **<0.0001** | **0.0043** |
| Optic Nerve L | IMRT | D95% | **0.0040** | **0.0006** | **0.0008** |
| Optic Nerve L | IMRT | D75% | **0.0002** | **<0.0001** | **0.0014** |
| Optic Nerve L | IMRT | D50% | **0.0020** | **0.0005** | **0.0018** |
| Optic Nerve L | IMRT | D25% | **0.0026** | **<0.0001** | **0.0090** |
| Optic Nerve L | IMRT | D5% | **0.0033** | **0.0005** | **0.0119** |
| Optic Nerve L | IMRT | D2% | **0.0031** | **0.0005** | **0.0095** |
| Optic Nerve L | IMPT | D95% | 1.0000 | 0.0508 | 0.2037 |
| Optic Nerve R | IMRT | Dmax | **0.0090** | **0.0010** | **0.0054** |
| Optic Nerve R | IMRT | Dmean | **0.0047** | **<0.0001** | **0.0012** |
| Optic Nerve R | IMRT | D95% | **0.0036** | **<0.0001** | **0.0066** |
| Optic Nerve R | IMRT | D75% | **0.0010** | **<0.0001** | **0.0038** |
| Optic Nerve R | IMRT | D50% | **0.0103** | **<0.0001** | **0.0013** |
| Optic Nerve R | IMRT | D25% | **0.0366** | **0.0003** | **0.0022** |
| Optic Nerve R | IMRT | D5% | **0.0035** | **0.0001** | **0.0284** |
| Optic Nerve R | IMRT | D2% | **0.0019** | **0.0006** | **0.0180** |
| Optic Nerve R | IMPT | D95% | 0.3264 | 0.2498 | 0.1181 |
| Parotid L | IMRT | Dmax | 1.0000 | 0.0719 | **0.0321** |
| Parotid L | IMRT | Dmean | 0.0756 | 0.3935 | 1.0000 |
| Parotid L | IMRT | D95% | **0.0030** | **0.0001** | **0.0043** |
| Parotid L | IMRT | D75% | **0.0408** | **0.0026** | **0.0043** |
| Parotid L | IMRT | D50% | 0.1324 | **0.0110** | **0.0219** |
| Parotid L | IMRT | D5% | **0.0321** | **0.0036** | 0.1748 |
| Parotid L | IMPT | D75% | **0.0110** | 0.5525 | 1.0000 |
| Parotid R | IMRT | Dmean | **0.0186** | 0.0645 | 1.0000 |
| Parotid R | IMRT | D95% | 0.2276 | **0.0128** | **0.0283** |
| Parotid R | IMRT | D75% | 0.1200 | **0.0127** | **0.0060** |
| Parotid R | IMRT | D50% | 0.5135 | **0.0081** | **0.0043** |
| Parotid R | IMRT | D25% | 0.0799 | **0.0051** | 0.0588 |
| Parotid R | IMRT | D5% | **0.0219** | **0.0110** | 0.0799 |
| Parotid R | IMPT | D95% | **0.0086** | **0.0305** | 1.0000 |
| Parotid R | IMPT | D75% | **0.0167** | 0.0887 | 0.9899 |
| Parotid R | IMPT | D50% | 0.1912 | **0.0249** | 0.5306 |
| Parotid R | IMPT | D5% | **0.0165** | **0.0321** | 1.0000 |
| Spinal Cord | IMRT | Dmean | **0.0026** | **0.0003** | **0.0004** |
| Spinal Cord | IMRT | D95% | **0.0008** | **0.0006** | **0.0009** |
| Spinal Cord | IMRT | D75% | 0.5525 | 0.1087 | 0.0577 |
| Spinal Cord | IMRT | D50% | **0.0127** | **0.0005** | **0.0002** |
| Spinal Cord | IMRT | D25% | 1.0000 | **0.0459** | **0.0127** |
| Spinal Cord | IMPT | Dmax | 0.1326 | 0.6485 | 1.0000 |
| Spinal Cord | IMPT | Dmean | 0.8828 | 0.0577 | **0.0321** |
| Spinal Cord | IMPT | D95% | 0.0502 | **0.0148** | 0.2206 |
| Spinal Cord | IMPT | D75% | 0.0693 | **0.0030** | **0.0068** |
| Large Arteries | IMRT | Dmean | **<0.0001** | **<0.0001** | **<0.0001** |
| Large Arteries | IMRT | D95% | **<0.0001** | **<0.0001** | **<0.0001** |
| Large Arteries | IMRT | D75% | **<0.0001** | **<0.0001** | **<0.0001** |
| Large Arteries | IMRT | D50% | **<0.0001** | **<0.0001** | **<0.0001** |
| Large Arteries | IMRT | D25% | **<0.0001** | **0.0004** | **<0.0001** |
| Large Arteries | IMRT | D5% | **0.0002** | **<0.0001** | **<0.0001** |
| Large Arteries | IMRT | D2% | **0.0008** | **<0.0001** | **<0.0001** |
| Large Arteries | IMPT | Dmax | 0.1748 | **0.0219** | 1.0000 |
| Large Arteries | IMPT | Dmean | **<0.0001** | **<0.0001** | **<0.0001** |
| Large Arteries | IMPT | D95% | **0.0051** | **0.0029** | **0.0067** |
| Large Arteries | IMPT | D75% | **<0.0001** | **<0.0001** | **<0.0001** |
| Large Arteries | IMPT | D50% | **<0.0001** | **<0.0001** | **<0.0001** |
| Large Arteries | IMPT | D25% | **<0.0001** | **<0.0001** | **0.0001** |
| Large Arteries | IMPT | D5% | **0.0008** | **0.0019** | 0.1453 |
| Large Arteries | IMPT | D2% | **<0.0001** | **<0.0001** | 0.1200 |
| Large Veins | IMRT | Dmax | 0.8828 | **0.0008** | **0.0167** |
| Large Veins | IMRT | Dmean | **<0.0001** | **<0.0001** | **<0.0001** |
| Large Veins | IMRT | D95% | **<0.0001** | **<0.0001** | **<0.0001** |
| Large Veins | IMRT | D75% | **<0.0001** | **<0.0001** | **<0.0001** |
| Large Veins | IMRT | D50% | **<0.0001** | **<0.0001** | **<0.0001** |
| Large Veins | IMRT | D25% | **<0.0001** | **<0.0001** | **<0.0001** |
| Large Veins | IMRT | D5% | **<0.0001** | **<0.0001** | **<0.0001** |
| Large Veins | IMRT | D2% | **<0.0001** | **<0.0001** | **<0.0001** |
| Large Veins | IMPT | Dmax | 1.0000 | **0.0321** | 0.7365 |
| Large Veins | IMPT | Dmean | **<0.0001** | **<0.0001** | **<0.0001** |
| Large Veins | IMPT | D95% | **0.0066** | **0.0044** | 0.8526 |
| Large Veins | IMPT | D75% | **0.0004** | **<0.0001** | **<0.0001** |
| Large Veins | IMPT | D50% | **<0.0001** | **<0.0001** | **0.0004** |
| Large Veins | IMPT | D25% | **<0.0001** | **<0.0001** | **0.0039** |
| Large Veins | IMPT | D5% | **0.0003** | **<0.0001** | **0.0005** |
| Large Veins | IMPT | D2% | **<0.0001** | **<0.0001** | **0.0167** |

Wilcoxon signed-rank test, Bonferroni-corrected p-values.

Abbreviations: BVS, blood vessel-sparing plan; Conv, conventional plan; D_max_, maximum dose; D_mean_, mean dose; D_n%_, The minimum dose received by at least n% of the blood volume; IMRT, intensity-modulated radiation therapy; IMPT, intensity-modulated proton therapy; OAR, organ-at-risk.

Table A2. Blood dose-volume histogram metrics of each treatment plan

| **Modality** | **Metric** | **Conv** | **BVS-90%** | **BVS-80%** | **Friedman p** | **Conv vs BVS-90%** | **Conv vs BVS-80%** | **BVS-90% vs BVS-80%** |
| --- | --- | --- | --- | --- | --- | --- | --- | --- |
| **IMRT** | Dmean | 1.4820 (1.0869–16.4832) | 1.3789 (1.0006–16.4572) | 1.3083 (0.9152–16.4464) | **<0.0001** | **<0.0001** | **<0.0001** | **0.0006** |
|  | D2% | 1.9280 (1.3024–17.8472) | 1.7543 (1.2073–17.8298) | 1.6778 (1.0517–17.8195) | **<0.0001** | **<0.0001** | **<0.0001** | **0.0021** |
|  | D10% | 1.7613 (1.2172–17.3275) | 1.5974 (1.1221–17.3044) | 1.5473 (0.9967–17.2953) | **<0.0001** | **<0.0001** | **<0.0001** | **0.0021** |
|  | D30% | 1.5812 (1.1379–16.8217) | 1.4706 (1.0447–16.7970) | 1.3945 (0.9461–16.7873) | **<0.0001** | **<0.0001** | **<0.0001** | **0.0012** |
|  | D50% | 1.4677 (1.0799–16.4751) | 1.3673 (0.9950–16.4505) | 1.2951 (0.9127–16.4406) | **<0.0001** | **<0.0001** | **<0.0001** | **0.0006** |
|  | D70% | 1.3870 (1.0255–16.1351) | 1.2885 (0.9483–16.1084) | 1.2140 (0.8808–16.0959) | **<0.0001** | **<0.0001** | **<0.0001** | **<0.0001** |
|  | D90% | 1.2842 (0.9541–15.6495) | 1.1904 (0.8866–15.6188) | 1.1183 (0.8369–15.6049) | **<0.0001** | **<0.0001** | **<0.0001** | **<0.0001** |
| **IMPT** | Dmean | 1.1976 (0.6836–16.2352) | 1.1014 (0.6565–16.2040) | 1.0587 (0.5715–16.2184) | **<0.0001** | **<0.0001** | **<0.0001** | **0.0146** |
|  | D2% | 1.9085 (0.9576–18.6462) | 1.7536 (0.9045–18.6224) | 1.6635 (0.7825–18.6449) | **<0.0001** | **<0.0001** | **<0.0001** | **0.0167** |
|  | D10% | 1.5845 (0.8388–17.7168) | 1.4575 (0.7977–17.6872) | 1.4111 (0.6921–17.7072) | **<0.0001** | **<0.0001** | **<0.0001** | **0.0192** |
|  | D30% | 1.3214 (0.7354–16.8238) | 1.2138 (0.7043–16.7938) | 1.1700 (0.6128–16.8126) | **<0.0001** | **<0.0001** | **<0.0001** | **0.0167** |
|  | D50% | 1.1625 (0.6716–16.2179) | 1.0696 (0.6463–16.1871) | 1.0290 (0.5634–16.1997) | **<0.0001** | **<0.0001** | **<0.0001** | **0.0127** |
|  | D70% | 1.0226 (0.6146–15.6227) | 0.9429 (0.5938–15.5889) | 0.9088 (0.5185–15.5993) | **<0.0001** | **<0.0001** | **<0.0001** | **0.0110** |
|  | D90% | 0.8622 (0.5441–14.7738) | 0.7991 (0.5286–14.7429) | 0.7657 (0.4617–14.7506) | **<0.0001** | **<0.0001** | **<0.0001** | **0.0095** |

Friedman test with post-hoc Wilcoxon signed-rank tests (Bonferroni-corrected, n=20). Values are median (range) in Gy. Bold p-values indicate p < 0.05.

Abbreviations: BVS, blood vessel-sparing plan; Conv, conventional plan; D_mean_, mean dose; D_n%_, The minimum dose received by at least n% of the blood volume; IMRT, intensity-modulated radiation therapy; IMPT, intensity-modulated proton therapy.

Table A3. Dice similarity coefficients of major blood vessels automatically contoured by RayStation and TotalSegmentator

| Structure | Dice similarity coefficient, median (range) |
| --- | --- |
| aorta | 0.917 (0.818 – 0.946) |
| large arteries^*^ | 0.641 (0.524 – 0.696) |
| large veins^**^ | 0.774 (0.393 – 0.817) |
| superior vena cava | 0.841 (0.743 – 0.904) |

* large arteries consist of common carotid arteries, subclavian arteries, and brachiocephalic trunk.

** large veins consist of brachiocephalic veins.

Table A4. Dosimetric comparison of large vessel contoured using TotalSegmentator vs RayStation

| **Structure** | **Plan** | **TotalSegmentation, median (range)** | **RayStation, median (range)** | **p-value** |
| --- | --- | --- | --- | --- |
| Large arteries | IMRT-Conv | 36.55 (27.17-52.29) | 33.60 (28.41-54.60) | 0.5205 |
|  | IMRT-BVS-90% | 30.84 (22.93-51.78) | 30.53 (25.19-54.23) | 0.7913 |
|  | IMRT-BVS-80% | 26.57 (20.89-47.26) | 27.70 (22.97-52.50) | 0.4274 |
|  | IMPT-Conv | 35.13 (26.74-50.48) | 32.11 (26.64-54.72) | 0.7337 |
|  | IMPT-BVS-90% | 30.08 (22.65-44.37) | 29.02 (23.69-53.21) | 0.9698 |
|  | IMPT-BVS-80% | 25.20 (20.93-41.63) | 27.17 (23.28-52.58) | 0.3447 |
| Large veins | IMRT-Conv | 23.69 (2.93-52.07) | 23.98 (3.38-52.23) | 0.8205 |
|  | IMRT-BVS-90% | 18.96 (2.47-48.43) | 21.66 (2.73-48.70) | 0.6776 |
|  | IMRT-BVS-80% | 17.45 (2.19-45.14) | 19.84 (2.47-46.26) | 0.7337 |
|  | IMPT-Conv | 22.92 (1.10-52.35) | 23.20 (2.19-52.50) | 0.7336 |
|  | IMPT-BVS-90% | 18.74 (0.54-49.68) | 20.91 (1.05-49.94) | 0.6232 |
|  | IMPT-BVS-80% | 14.24 (0.50-49.10) | 18.76 (0.93-49.47) | 0.6775 |

Mean dose comparison with Mann-Whitney U test.

Supplementary section A1.

Incorporating blood dose simulations into the dose optimization during RT treatment planning directly is a time-consuming process. Ultimately, it is essential to develop an approach in which blood dose can be accurately and rapidly estimated using either an analytical or AI-based model, allowing for its seamless integration into the cost function of the optimization. However, even without precise modeling, a reasonable approximation of blood dose—albeit with some degree of uncertainty—can still contribute to effective plan optimization. In this study, we adopted a strategy that reduces blood dose by imposing dose constraints on large arteries and veins. That is, changes of dose to large vessels may serve as a surrogate metric for estimating the corresponding changes in blood dose.

This section proposes a practical approach for rapidly estimating blood dose changes. Indeed, blood dose estimation is highly dependent on planning parameters, tumor location, and treatment modality. The formulated polynomial equations for IMRT and IMPT are derived based on representative dosimetric parameters and fitted to dose outcomes planned in this study.

$${\Delta D}_{blood}=a{\Delta D}_{large arteries}+b\Delta D_{large veins}+cV_{PTV1}+dV_{PTV2}+e$$

Where $a$, $b$, $c$, $d$, and $e$ represent the coefficients of the respective independent variables and the intercept. $\Delta D_{structure}$ and $V_{structure}$ denote the dose change and volume of the corresponding structure, respectively. The fitted coefficients and intercept values, as detailed in Table 5, reflect the relationship between these parameters and blood dose, enabling a rapid yet reasonable estimation of blood dose reduction based on large vessel-sparing. The adjusted R-squared scores were 0.713 for IMRT and 0.721 for IMPT. Although this relationship is influenced by tumor location, treatment technique, and planning parameters, it holds promising potential for clinical application, particularly in standardized treatment protocols for which routine plan optimization is performed.

Table A5-1. Comparison between calculated and predicted blood dose in IMPT

| Case | Calculated dose change [Gy] | Predicted dose change [Gy] | Error rate [%] |
| --- | --- | --- | --- |
| 1 | 0.104 | 0.156 | 33.247 |
| 2 | 0.112 | 0.133 | 15.895 |
| 3 | 0.121 | 0.129 | 6.116 |
| 4 | 0.195 | 0.169 | 15.127 |
| 5 | 0.17 | 0.179 | 4.84 |
| 6 | 0.285 | 0.254 | 12.417 |
| 7 | 0.137 | 0.153 | 10.723 |
| 8 | 0.132 | 0.12 | 9.694 |
| 9 | 0.072 | 0.046 | 56.749 |
| 10 | 0.108 | 0.096 | 11.671 |

$\boldsymbol{a}$ = 0.017, $\boldsymbol{b}$ = 0.013, $\boldsymbol{c}$ = -0.041, $\boldsymbol{d}$ = -0.053, $\boldsymbol{e}$ = 0.236

Table A5-2. Comparison between calculated and predicted blood dose in IMRT

| Case | Calculated dose change [Gy] | Predicted dose change [Gy] | Error rate [%] |
| --- | --- | --- | --- |
| 1 | 0.214 | 0.229 | 6.653 |
| 2 | 0.181 | 0.181 | 0.367 |
| 3 | 0.154 | 0.175 | 11.778 |
| 4 | 0.207 | 0.2 | 3.294 |
| 5 | 0.212 | 0.225 | 5.879 |
| 6 | 0.252 | 0.237 | 6.592 |
| 7 | 0.218 | 0.203 | 7.255 |
| 8 | 0.186 | 0.187 | 0.463 |
| 9 | 0.185 | 0.173 | 6.793 |
| 10 | 0.161 | 0.159 | 1.177 |

$\boldsymbol{a}$ = 0.008, $\boldsymbol{b}$ = 0.015, $\boldsymbol{c}$ = -0.005, $\boldsymbol{d}$ = -0.006, $\boldsymbol{e}$ = 0.124
